# Incremental column-wise verification of arithmetic circuits using computer algebra

**DOI:** 10.1007/s10703-018-00329-2

**Published:** 2019-02-26

**Authors:** Daniela Kaufmann, Armin Biere, Manuel Kauers

**Affiliations:** 1 Institute for Formal Models and Verification, Johannes Kepler University, Linz, Austria; 2 Institute for Algebra, Johannes Kepler University, Linz, Austria

**Keywords:** Arithmetic circuit verification, Computer algebra, Gröbner basis

## Abstract

Verifying arithmetic circuits and most prominently multiplier circuits is an important problem which in practice still requires substantial manual effort. The currently most effective approach uses polynomial reasoning over pseudo boolean polynomials. In this approach a word-level specification is reduced by a Gröbner basis which is implied by the gate-level representation of the circuit. This reduction returns zero if and only if the circuit is correct. We give a rigorous formalization of this approach including soundness and completeness arguments. Furthermore we present a novel incremental column-wise technique to verify gate-level multipliers. This approach is further improved by extracting full- and half-adder constraints in the circuit which allows to rewrite and reduce the Gröbner basis. We also present a new technical theorem which allows to rewrite local parts of the Gröbner basis. Optimizing the Gröbner basis reduces computation time substantially. In addition we extend these algebraic techniques to verify the equivalence of bit-level multipliers without using a word-level specification. Our experiments show that regular multipliers can be verified efficiently by using off-the-shelf computer algebra tools, while more complex and optimized multipliers require more sophisticated techniques. We discuss in detail our complete verification approach including all optimizations.

## Introduction

Formal verification of arithmetic circuits is important to help to prevent issues like the famous Pentimum FDIV bug. Even more than 20 years after detecting this bug the problem of verifying arithmetic circuits and especially multiplier circuits is still considered to be hard. A common approach models the verification problem as a satisfiability (SAT) problem, in which the circuit is translated into a formula in conjunctive normal form (CNF) which is then passed on to SAT-solvers. In order to stimulate the development of fast SAT solving techniques for arithmetic circuit verification, a large set of these benchmarks was generated and the CNF encodings were submitted to the SAT 2016 competition. They are publicly available [4]. The competition results confirmed that miters of even small multipliers produce very hard SAT problems. The weak performance of SAT solvers on this benchmark set lead to the conjecture that verifying miters of multipliers and other ring properties after encoding them into CNF needs exponential sized resolution proofs [6], which would imply exponential run-time of CDCL SAT solvers. However, this conjecture was recently rebutted. In [2] it was shown that such ring properties do admit polynomial sized resolution proofs. But since proof search is non-deterministic, this theoretical result still needs to be transferred into practical SAT solving.

Alternative verification techniques use decision diagrams [9, 10], more specifically binary decision diagrams (BDDs) and binary moment diagrams (BMDs) are used for circuit verification. The drawback of BDDs is their high usage of memory for this kind of benchmarks [9]. This issue can be resolved by using BMDs which remain linear in the number of input bits of a multiplier. Actually BMDs and variants of them have been shown to be capable of detecting the Pentium FDIV bug. However, the BMD approach is not robust, it still requires explicit structural knowledge of the multipliers [14]. It is important to determine the order in which BMDs are built, because it has tremendous influence on performance. Actually only a row-wise backward substitution approach seems to be feasible [13], which in addition assumes a simple carry-save-adder (CSA) design.

The currently most effective approach for gate-level verification of arithmetic circuits uses computer algebra [15, 24, 27–31, 35]. For each gate in the circuit a polynomial is introduced which represents the relation of the gate output and the inputs of the gate. To ensure that variables in the circuit are restricted to boolean values, additional so-called “field polynomials” are introduced. Furthermore the word-level specification of the multiplier is modeled as a polynomial. If the circuit variables are ordered according to their reverse topological appearance in the circuit, i.e., a gate output variable is greater than the input variables of the gate, then the gate polynomials and field polynomials form a Gröbner basis. As a consequence, the question if a gate-level circuit implements a correct multiplier can be answered by reducing the multiplier specification polynomial by the circuit Gröbner basis. The multiplier is correct if and only if the reduction returns zero.

Related work [15, 35] uses a similar algebraic approach, which is called function extraction. The word-level output of the circuit is rewritten using the gate relations and the goal is to derive a unique polynomial representation of the gate inputs. In order to verify correctness of the circuit this polynomial is then compared to the circuit specification. This rewriting method is essentially the same as Gröbner basis reduction and is able to handle very large clean multipliers but fails on slightly optimized multiplier architectures. The authors of [24, 27, 37] focus on verification of Galois field multipliers using Gröbner basis theory. In contrast we focus in our work [8, 28, 29] on integer multipliers as the authors of [15, 30, 31, 35] do. In [30, 31] the authors propose a sophisticated reduction scheme which is used to rewrite and simplify the Gröbner basis, which as a consequence reduces computation time substantially. Several optimizations are introduced which made their verification technique scale to large multipliers of various architectures [19], but their arguments for soundness and completeness are rather imprecise and neither the tools nor details about experiments are publicly available.

Inspired by these ideas we presented in [28] an incremental column-wise verification technique for integer multipliers where a multiplier circuit is decomposed into columns. In each column the partial products can be uniquely identified and we can define a distinct specification for each slice relating the partial products, incoming carries, slice output and outgoing carries of the slice. We incrementally apply Gröbner basis reduction on the slices to verify the circuit. The incremental column-wise checking algorithm is improved in [8, 29]. The idea in this work is to simplify the Gröbner basis by introducing linear adder specifications. We search for full- and half-adder structures in the gate-level circuit and eliminate the internal gates of the adder structures, with the effect of reducing the number of polynomials in the Gröbner basis. Furthermore we are able to include adder specifications in the Gröbner basis. Reducing by these linear polynomials leads to substantial improvements in terms of computation time.

Alternatively to circuit verification using a word-level specification, it is also common to check the equivalence of a gate-level circuit and a given reference circuit. This technique is extremely important when it is not possible to write down the word-level specification of a circuit in a canonical expression. In [32] equivalence checking of multiplier circuits is achieved by first extracting half-adder circuits from the accumulation of partial products and then checking the equivalence of these extracted half-adder circuits. Proofs of soundness and completeness are lacking. More recently [31] proposes an algebraic variant of combinational equivalence checking based on Gröbner basis theory. It is similar to SAT sweeping [23], and compares the circuits bit-wise, e.g., output bit by output bit, again without soundness nor completeness proof.

As a further contribution we present an extension of our incremental column-wise verification approach, which can be used to incrementally derive the equivalence of two arbitrary gate-level circuits in a column-wise fashion. We prove soundness and completeness for this method.

This article extends and revises work presented earlier in [8, 28, 29]. Extending [28], we provide a more detailed description of the algebraic approach, including several examples. In Sect. 4 we introduce additional rewriting methods, called “Partial Product Elimination” and “Adder-Rewriting” [8, 29], which help to further simplify the Gröbner basis. We present the theory behind these rewriting approaches in Sect. 5 including a theoretical theorem [8], which allows that only a local part of the Gröbner basis is rewritten without losing the Gröbner basis property. In Sect. 8 we generalize our incremental column-wise verification approach to an incremental equivalence checking approach [29].

For this article we revised our engineering techniques and discuss a new method to derive our column-wise slices in Sect. 9, which reduces the need of reallocating gates. Furthermore we were able to improve the computation time of the experiments in [28] by adjusting the order of polynomials during printing, cf. Sect. 9.

## Algebra

Following [8, 15, 24, 27–31, 35], we model the behavior of a circuit using multivariate polynomials. For each input and output of a logical gate a variable is introduced. The behavior of a gate, i.e., the relation of the gate inputs to the output of a gate is translated into a polynomial. The set of all these polynomials builds a comprehensive description of the circuit. We show that the circuit is correct if and only if the circuit specification, a polynomial describing the relation of the circuit inputs and outputs, is implied by the gate-level polynomials.

The appropriate formalism for such a reasoning is the theory of Gröbner bases [11, 12, 16]. Throughout this section let $\mathbb {K}[X]=\mathbb {K}[x_1,\dots ,x_n]$ denote the ring of polynomials in variables $x_1,\dots ,x_n$ with coefficients in the field $\mathbb {K}$.

### Definition 1

A *term* (or *power product*) is a product of the form $x_1^{e_1}\cdots x_n^{e_n}$ for certain non-negative exponents $e_1,\dots ,e_n\in \mathbb {N}$. The set of all terms is denoted by [*X*]. A *monomial* is a constant multiple of a term, $\alpha x_1^{e_1}\cdots x_n^{e_n}$ with $\alpha \in \mathbb {K}$. A *polynomial* is a finite sum of monomials.

On the set of terms we fix an order such that for all terms $\tau ,\sigma _1,\sigma _2$ we have $1\le \tau$ and $\sigma _1\le \sigma _2\Rightarrow \tau \sigma _1\le \tau \sigma _2$. Such an order is called a *lexicographic term order* if for all terms $\sigma _1= x_1^{u_1}\cdots x_n^{u_n}$, $\sigma _2= x_1^{v_1}\cdots x_n^{v_n}$ we have $\sigma _1 < \sigma _2$ iff there exists an index *i* with $u_j = v_j$ for all $j<i$, and $u_i<v_i$.

Since every polynomial $p\ne 0$ contains only finitely many terms and they are ordered according to our fixed order <, we can determine the largest term in a polynomial. We call it the *leading term* of *p* and write ${\text {lt}}(p)$. If $p=c\tau + \cdots$ and ${\text {lt}}(p)=\tau$, then ${\text {lc}}(p)=c$ is called the *leading coefficient* and ${\text {lm}}(p)=c\tau$ is called the *leading monomial* of *p*. The *tail* of *p* is defined by $p-c\tau$.

### Definition 2

A nonempty subset $I\subseteq \mathbb {K}[X]$ is called an *ideal* if

$$\begin{aligned} \forall \ p,q\in I: p+q\in I \quad \text { and }\quad \forall \ p\in \mathbb {K}[X]\ \forall \ q\in I: pq\in I. \end{aligned}$$

If $I\subseteq \mathbb {K}[X]$ is an ideal, then a set $P=\{p_1,\dots ,p_m\}\subseteq \mathbb {K}[X]$ is called a *basis* of *I* if $I=\{q_1p_1+\cdots +q_mp_m\mid q_1,\dots ,q_m\in \mathbb {K}[X]\}$, i.e., if *I* consists of all the linear combinations of the $p_i$ with polynomial coefficients. We denote this by $I= \langle P \rangle$ and say *I* is generated by *P*.

In general, an ideal *I* has many bases which generate the ideal. We are particularly interested in bases with certain structural properties, called Gröbner bases.

### Definition 3

A basis $G=\{g_1,\dots ,g_n\}$ of an ideal $I\subseteq \mathbb {K}[X]$ is called a *Gröbner basis* (w.r.t. the fixed order $\le$) if the leading term of every nonzero element of *I* is a multiple of (at least) one of the leading terms ${\text {lt}}(g_1),\dots ,{\text {lt}}(g_n)$.

### Lemma 1

Every ideal $I \subseteq \mathbb {K}[X]$ has a Gröbner basis w.r.t. a fixed term order.

### Proof

Corollary 6 in Chap. 2 §5 of  [16]. $\square$

The following Lemma 2 describes *Buchberger’s Criterion*, which states when a basis of an ideal is a Gröbner basis. Given an arbitrary basis of an ideal, Buchberger’s algorithm [11] is able to compute a Gröbner basis for it in finitely many steps. The algorithm is based on repeated computation of so-called S-polynomials.

### Lemma 2

Let $G\subseteq \mathbb {K}[X]{\setminus }\{0\}$ be a basis of an ideal $I =\langle G\rangle$. We define *S-polynomials*

$$\begin{aligned} {\text {spol}}(p,q)\,:=\,{\text {lcm}}({\text {lt}}(p),{\text {lt}}(q))\biggl (\frac{p}{{\text {lm}}(p)}- \frac{q}{{\text {lm}}(q)}\biggr ) \end{aligned}$$

for all $p,q\in \mathbb {K}[X]{\setminus }\{0\}$, with ${\text {lcm}}$ the least common multiple. Then *G* is a Gröbner basis of the ideal *I* if and only if the remainder of the division of ${\text {spol}}(p,q)$ by *G* is zero for all pairs $(p,q)\in G\times G$.

### Proof

Thm. 6 in Chap. 2 §6 of [16]. $\square$

To reduce the computation effort of Buchberger’s algorithm several optimizations exist which decrease the number of S-polynomial computations. We will heavily make use of the following optimization.

### Lemma 3

(Product criterion) If $p,q\in \mathbb {K}[X]{\setminus }\{0\}$ are such that the leading terms are coprime, i.e., ${\text {lcm}}({\text {lt}}(p),{\text {lt}}(q)) = {\text {lt}}(p){\text {lt}}(q)$, then ${\text {spol}}(p,q)$ reduces to zero mod $\{p,q\}$.

### Proof

Property 4 in Chap. 2 §9 of [16].$\square$

Since $\{p,q\}\subseteq G$, Lemma 3 suggests that if all leading terms of the polynomials in a basis *G* of an ideal *I* are coprime, i.e., we cannot find any pair of polynomials $p,q \in G$ such that ${\text {lt}}(p)$ and ${\text {lt}}(q)$ have any variable in common, then the product criterion holds for all pairs of polynomials of *G* and thus *G* is automatically a Gröbner basis for the ideal *I*.

To answer the question if a circuit is correct and hence fulfills its specification we need to check if the specification polynomial is contained in the ideal generated by the circuit relations, as we discuss in detail in Sect. 3. The theory of Gröbner bases offers a decision procedure for this so-called *ideal membership problem:* Given a polynomial $f\in \mathbb {K}[X]$ and an ideal $I= \langle G \rangle \subseteq \mathbb {K}[X]$, determine if $f \in I$.

Given an arbitrary basis *G* of the ideal *I*, it is not so obvious how to check whether the polynomial *f* belongs to the ideal $I = \langle G\rangle$. However, if *G* is a Gröbner basis of *I*, then the membership question can be answered using a multivariate version of polynomial division with remainder, cf. Algorithm 1, as derivation procedure. It can be shown that whenever *G* is a Gröbner basis, then *f* belongs to the ideal generated by *G* if and only if the remainder of division of *f* by *G* is zero. In the following we will introduce this approach more formally.

### Lemma 4

(Multivariate division with remainder) Let the set of terms be ordered according to a fixed order < and let $P = (p_1, \ldots , p_s)$ be an ordered list of polynomials in $\mathbb {K}[X]$. Then every $f \in \mathbb {K}[X]$ can be written as:

$$\begin{aligned} f = h_1p_1 + \ldots + h_sp_s + r \end{aligned}$$

where $h_1,\ldots ,h_s, r \in \mathbb {K}[X]$. The remainder *r* is either zero or is a polynomial $\in \mathbb {K}[X]$, such that no term in *r* is a multiple of some ${\text {lt}}(p_i)$. The complete division algorithm is listed in Algorithm 1. We call the polynomials $h_i$ the *co-factors* of *f* and the polynomial *r* is called the *remainder of f with respect to P*.

### Proof

Thm. 3 in Chap. 2 §3 of [16].$\square$


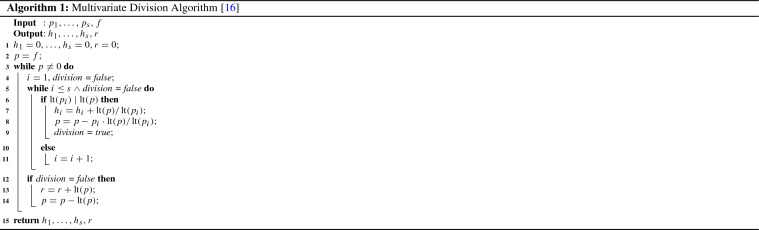


### Example 1

**Fig. 1:**
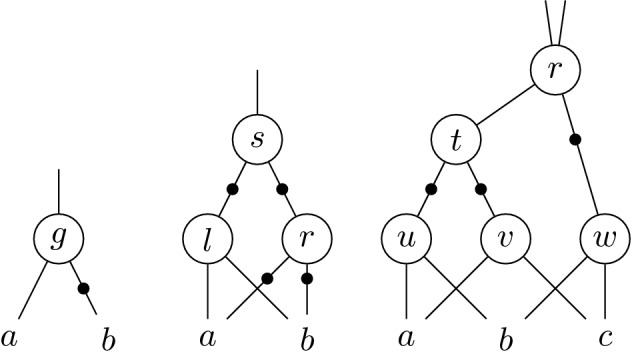
And-Inverter Graphs (AIGs) [23] used in Example 1 and later in Sect. 4

Figure 1 depicts several And-Inverter-Graphs (AIGs) [23]. A node in an AIG represents logical conjunction of the two inputs, depicted by edges on the lower half of the node. The output is depicted by an edge in the upper half of the node. An edge containing a marker negates the variable.

Let $\mathbb {K} = \mathbb {Q}.$ Hence for the AIG on the left of Fig. 1, we have the relation $g=a(1-b)$ for all $a,b,g\in \{0,1\}$. Furthermore, we always have $g(g-1)=a(a-1)=b(b-1)=0$ since $a,b,g\in \{0,1\}$. To show that we always have $gb=0$, it suffices to check if the polynomial $gb\in \mathbb {Q}[g,a,b]$ is contained in the ideal $I\subseteq \mathbb {Q}[g,a,b]$ with

$$\begin{aligned} I=\langle -g+a(1-b),~g(g-1),~a(a-1),~b(b-1)\rangle . \end{aligned}$$

Multivariate polynomial division yieldswith $h_2=h_3=0$, and therefore $gb\in I$ and thus $gb=0$ in the left AIG of Fig. 1.

As shown in this example, we can view an ideal $I=\langle G \rangle \subseteq \mathbb {K}[X]$ as an equational theory, where the basis $G=\{g_1,\dots ,g_m\}$ defines the set of axioms. The ideal $I = \langle G \rangle$ contains exactly those polynomials *f* for which the equation “$f=0$” can be derived from the axioms “$g_1=\cdots =g_m=0$” through repeated application of the rules $u=0\wedge v=0\Rightarrow u+v=0$ and $u=0\Rightarrow uw=0$ (compare to Definition 2).

### Lemma 5

If $G = \{g_1,\ldots ,g_m\}$ is a Gröbner basis, then every $f\in \mathbb {K}[X]$ has a unique remainder *r* with respect to *G*. Furthermore it holds that $f-r \in \langle G \rangle$.

### Proof

Property 1 in Chap. 2 §6 of [16].$\square$

Ultimately the following Lemma provides the answer on how we can solve the ideal membership problem with the help of Gröbner basis and thus can check whether a polynomial belongs to an ideal or not.

### Lemma 6

Let $G = \{g_1,\ldots ,g_m\}\subseteq \mathbb {K}[X]$ be a Gröbner basis, and let $f\in \mathbb {K}[X]$. Then *f* is contained in the ideal $I=\langle G\rangle$ iff the remainder of *f* with respect to *G* is zero.

### Proof

Corollary 2 in Chap. 2 §6 of [16].$\square$

## Ideals associated to circuits

We consider circuits *C* with two bit-vectors $a_0,\dots ,a_{n-1}$ and $b_0,\dots ,b_{n-1}$ of size *n* as inputs, and a bit-vector $s_0,\dots ,s_{2n-1}$ of size 2*n* as output. The circuit is represented by a number of logical gates where the output of some gate may be input to some other gate, but cycles in the circuit are not allowed. Additionally to the variables $a_i, b_i, s_i$ for the inputs and outputs of the circuit, we associate a variable $g_1,\dots ,g_k$ to each internal gate output. In our setting let $\mathbb {K} = \mathbb {Q}$. By *R* we denote the ring $\mathbb {Q}[a_0,\dots ,a_{n-1},b_0,\dots ,b_{n-1},g_1,\dots ,g_k,s_0,\dots ,s_{2n-1}]$, containing all polynomials in the above variables with coefficients in $\mathbb {Q}$. At first glance it may seem surprising that we use $\mathbb {Q}$ instead of $\mathbb {Z}_2$ as ground field although all our variables are restricted to boolean values. The reason for this choice is that we want to verify correctness of integer multiplication. As we will see in Definition 5, using $\mathbb {Q}$ as base field allows us to describe the desired behavior of the circuit by connecting it to the multiplication in $\mathbb {Q}$. It would also be possible to use $\mathbb {Z}_2$, but in this case, specifying the desired behavior of the circuit in terms of polynomial equations would not be much easier than constructing a circuit in the first place. Such a specification would not be more trustworthy than the circuit that we want to verify.

The semantic of each circuit gate implies a polynomial relation among the input and output variables, such as the following ones:

1 $$\begin{aligned} \begin{array}{lcl} u=\lnot v &{}\quad \text{ implies }&{}\quad 0= -u + 1 -v \\ u=v \wedge w &{}\quad \text{ implies } &{}\quad 0=-u + vw \\ u=v \vee w &{}\quad \text{ implies } &{}\quad 0=-u + v + w - vw \\ u=v \oplus w &{}\quad \text{ implies } &{}\quad 0=-u + v + w - 2vw. \end{array} \end{aligned}$$

The polynomials in *R* are chosen such that the boolean roots of the polynomials are the solutions of the corresponding gate constraints and vice versa. We denote these polynomials by *gate polynomials*. To ensure that we only find boolean solutions of the polynomials we add the relations $u(u-1)=0$ for each variable *u*. We call this relations *field polynomials*.

### Example 2

**Fig. 2:**
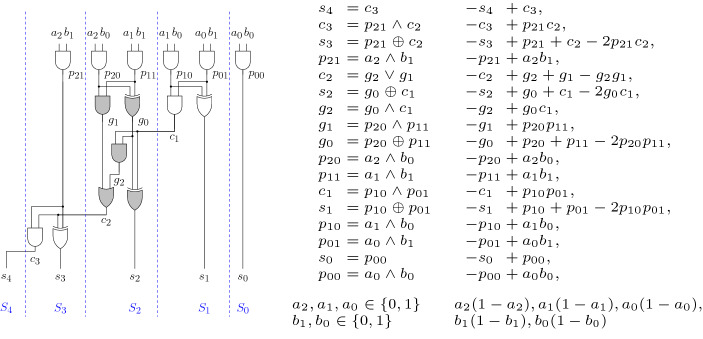
A $3\times 2$-bit gate-level multiplier circuit, gate constraints, and polynomials. Colored gates represent a full adder, cf. Sect. 5. Dashed lines depict column-wise slicing, cf. Sect. 7

The possible boolean solutions for the gate constraint $p_{00} = a_0 \wedge b_0$ of Fig. 2 represented as $(p_{00},a_0,b_0)$ are (1, 1, 1), (0, 1, 0), (0, 0, 1), (0, 0, 0) which are all solutions of the polynomial $-p_{00} + a_0b_0=0$, when $a_0, b_0$ are restricted to the boolean domain.

Since the logical gates in a circuit are functional, the values of all the variables $g_1,\dots ,g_k,s_0,\dots ,s_{2n-1}$ in a circuit are uniquely determined as soon as the inputs $a_0,\dots ,a_{n-1},b_0,\dots ,b_{n-1}\in \{0,1\}$ are fixed. This motivates the following definition.

### Definition 4

Let *C* be a circuit. A polynomial $p\in R$ is called a *polynomial circuit constraint (PCC)* for *C* if for every choice of

$$\begin{aligned} (a_0,\dots ,a_{n-1},b_0,\dots ,b_{n-1})\in \{0,1\}^{2n} \end{aligned}$$

and the resulting values $g_1,\dots ,g_k,s_0,\dots ,s_{2n-1}$ which are implied by the gates of the circuit *C*, the substitution of all these values into the polynomial *p* gives zero. The set of all PCCs for *C* is denoted by *I*(*C*).

It can easily be verified that *I*(*C*) is an ideal of *R*. Since it contains all PCCs, this ideal includes all relations that hold among the values at the different points in the circuit. Therefore, the circuit fulfills a certain specification if and only if the polynomial relation corresponding to the specification of the circuit is contained in the ideal *I*(*C*).

### Definition 5

A circuit *C* is called a *multiplier* if the word-level specification

$$\begin{aligned} \sum _{i=0}^{2n-1} 2^i s_i - \left( \sum _{i=0}^{n-1} 2^i a_i\right) \left( \sum _{i=0}^{n-1} 2^i b_i\right) \;\in \; I(C). \end{aligned}$$

Thus checking whether a given circuit *C* is a correct multiplier reduces to an ideal membership test. Definition 4 does not provide any information of a basis of *I*(*C*), hence Gröbner basis technology is not directly applicable. However, we can deduce at least some elements of *I*(*C*) from the semantics of circuit gates.

### Definition 6

Let *C* be a circuit. Let $G\subseteq R$ be the set which contains for each gate of *C* the corresponding polynomial of Eq. 1, where the variable *u* is replaced by the output variable and *v*, *w* are replaced by the input variables of the gate. Furthermore *G* contains the polynomials $a_i(a_i-1)$ and $b_i(b_i-1)$ for $0 \le i < n$, called *input field polynomials*. Then the ideal $\langle G \rangle \subset R$ is denoted by *J*(*C*).

Hence *G* is a basis for the ideal *J*(*C*) and we can decide membership using Gröbner bases theory. Assume that we have a verifier which checks for a given circuit *C* and a given specification polynomial $p \in R$ if *p* is contained in the ideal *J*(*C*). Because it holds that $J(C)\subseteq I(C)$, such a verifier is sound. To show that the verifier is also complete, we further need to show $J(C)\supseteq I(C)$. For doing so, we recall an important observation shown for instance in [24, 33].

### Theorem 1

Let *C* be a circuit, and let *G* be as in Definition 6. Furthermore let $\le$ be a reverse topological lexicographic term order where the variables are ordered such that the variable of a gate output is always greater than the variables attached to the input edges of that gate. Then *G* is a Gröbner basis with respect to the ordering $\le$.

### Proof

By the restrictions on the term order and the form of Eq. 1, the leading term of each gate polynomial is simply the output variable of the corresponding gate. Furthermore, the leading terms of the input field polynomials $a_i(a_i-1)$ and $b_i(b_i-1)$ are $a_i^2$ and $b_i^2$. Hence all leading terms are coprime and therefore, by Lemma 3, division of ${\text {spol}}(p,q)$ by $\{p,q\}$ has remainder zero for any choice $p,q\in G$. Since $\{p,q\}\subseteq G$ for all $p,q\in G$, division of ${\text {spol}}(p,q)$ by *G* gives the remainder zero for all $p,q\in G$, and then, by Lemma 2, the claim follows. $\square$

### Theorem 2

For all acyclic circuits *C*, we have $J(C)=I(C)$.

### Proof

“$\subseteq$” (soundness): Immediately follows from the definition of *J*(*C*).

“$\supseteq$” (completeness): Let $p \in R$ be a polynomial with $p\in I(C)$. We show that $p\in J(C)$. Since *C* is acyclic, we can order the variables according to the needs of Theorem 1. Hence by Theorem 1 we can derive a Gröbner basis *G* for *J*(*C*). Let *r* be the remainder of division of *p* by *G*. Thus $p-r\in J(C)$ by Lemma 5, and $r\in J(C)\iff p\in J(C)$. Then, since $J(C) \subseteq I(C)$ it holds that $p-r\in I(C)$. By $p\in I(C)$ and $p-r\in I(C)$ it follows that $r\in I(C)$. Thus we need to show $r\in J(C)$.

By the choice of the ordering of the terms and the observations about the leading terms in *G* made in the proof of Theorem 1, from Lemma 5 it also follows that *r* only contains input variables $a_0,\dots ,a_{n-1},b_0,\dots ,b_{n-1}$, and each of them has a maximum degree of one. Simultaneously, $r\in I(C)$ implies that all evaluations of *r* for all choices $a_i,b_j\in \{0,1\}$ are zero.

We show $r=0$, and thus $r \in J(C)$. Assume $r\ne 0$. Suppose *m* is a monomial of *r* with a minimal number of variables, including the case that *m* is a constant. Since the exponents are at most one, no two monomials in *r* contain exactly the same variables. Now select $a_i$ ($b_j$) to evaluate to 1 iff $a_i \in m$ ($b_j \in m$). Hence all monomials of *r* except *m* evaluate to zero and thus vanish. By this choice *r* evaluates to the (non-zero) coefficient of *m*, contradicting $r\in I(C)$. Thus $r=0$. $\square$

### Example 3

In contrast to our definition of a circuit, where both input bit-vectors have the same length, Fig. 2 shows a $3 \times 2$-bit multiplier. The leading terms of the polynomials in the right column, read from top to bottom, follow a reverse topological lexicographic ordering. Hence these polynomials form a Gröbner basis.

We conclude this section with the following simple but important observations. First, the ideal *I*(*C*) is a so-called vanishing ideal. Therefore, it follows that *J*(*C*) is a radical ideal. Hence testing ideal membership of the specification is sufficient for verifying the correctness of a circuit, and we do not need to apply the stronger radical membership test (cf. Chap. 4 §2 of [16]).

Second, since it holds that $I(C)=J(C)$ contains all the *field polynomials* $u (u - 1)$ for all variables *u*, not only for the inputs, we may add them to *G*.

Third, in the Gröbner basis *G* for gate-level circuits defined as given in Definition 6 using Eq. 1 it holds that all polynomials have leading coefficient $\pm \,1$. Thus during reduction (division) no coefficient outside of $\mathbb {Z}$ (with non-trivial denominator) is introduced. Hence all coefficient computations actually remain in $\mathbb {Z}$. This formally shows that the implementations, e.g., those from [30, 35], used for proving ideal membership to verify properties of gate-level circuits, actually can rely on computation in $\mathbb {Z}$ without loosing soundness nor completeness. Of course it needs to hold that the same term order as in Theorem 1 is used.

Fourth, we do not need $\mathbb {Z}$ as coefficient ring if we use computer algebra systems, we can simply choose any field containing $\mathbb {Z}$, e.g., $\mathbb {Q}$, which actually improves computation, because $\mathbb {Z}$ is not a field and ideal theory over rings is harder than ideal theory over fields. In our experiments, using rational coefficients made a huge difference for Singular [17] (but did not show any effect in Mathematica [34]).

Fifth, because the leading terms of *G* contain only one variable, computing a remainder with respect to *G* has the same effect as substituting each leading term with the corresponding tail until no further substitution is possible.

Sixth, given a circuit *C*, checking whether an assignment of the inputs exists, which yields a certain value at an output is actually the same as (circuit) SAT, and hence is NP complete:

### Corollary 1

Consider the problem to decide, for a given polynomial $p\in \mathbb {Q}[X]$ and a given Gröbner basis $G\subseteq \mathbb {Q}[X]$, whether $p \in \langle G\rangle$. Taking the bit-size of *p* and *G* in the natural encoding as a measure for the problem size, this problem is co-NP-hard.

### Proof

Circuit SAT is the problem to decide for a given circuit with *n* gates and one output bit whether it produces the output 1 for at least one choice of inputs. This problem is known to be NP-hard. Consequently, the problem of deciding whether a given circuit with *n* gates and one output bit *s* produces the output 1 for every choice of inputs is co-NP-hard. A circuit *C* returns 1 for every input iff $s-1\in J(C)$. As the Gröbner basis *G* for the circuit *C* has essentially the same size as *C*, the circuit problem can be solved with at most polynomial overhead if we have an algorithm for solving the membership problem. $\square$

The main point of this corollary is not that ideal membership is difficult, but that it remains difficult even if we assume to be given a Gröbner basis of the ideal as part of the input. For other results on the complexity of the ideal membership problem, see [1, 21].

As a final remark, in the case when a polynomial *g* is not contained in an ideal $I=\langle G\rangle$, i.e., the remainder of dividing *g* by *G* is not zero, the last part in the proof of Theorem 2, where the “smallest” monomial *m* is evaluated, allows to determine a concrete choice of input assignments for which *g* does not vanish. In our application of multiplier verification these evaluations provide counter-examples, in case a circuit is determined not to be a multiplier.

We claim that this section shows the first formalization of not only soundness but also completeness arguments for recent successful algebraic approaches [30, 35]. In previous work soundness and completeness was formally shown too but only for other polynomial rings, i.e., over ${\mathbb {F}}_{2^q}$ to model circuits which implement Galois-field multipliers  [24, 27], or for polynomial rings over ${\mathbb {Z}}_{2^q}$ which model arithmetic circuit verification using overflow semantics [33]. In the work of [35] soundness and completeness is discussed too, but refers to [24, 27] instead of showing proofs, which as discussed above uses coefficients in ${\mathbb {F}}_{2^q}$ and not $\mathbb {Z}$, the coefficient ring the approach [35] is actually working with.

## Optimizations

In this section we extend the “XOR-Rewriting”, “Common-Rewriting” and “Vanishing Constraints” optimizations of [28] by the additional rewriting techniques of “Adder-Rewriting” and “Partial Product Elimination” [8, 29]. Picking up the statement of Corollary 1, simply reducing the specification polynomial in the constructed Gröbner basis of the circuit generally leads to an exponential number of monomials in the intermediate reduction results. This conjecture was also made in [30]. Thus in practice to efficiently use polynomial reduction for verification of specific circuits tailored heuristics which rewrite Gröbner bases and hence improve the reduction process become very important to speed up computation. The (non-reduced) Gröbner basis of an ideal is not unique, thus some Gröbner bases may be better than others, for instance much smaller. A natural choice among all the Gröbner bases is the unique reduced Gröbner basis [16], but it was shown empirically in [29] that the computation of this basis for multipliers is not feasible in practice, e.g., the computation of the unique reduced Gröbner basis for a 4-bit multiplier took more than 20 min.

In [30] a logic reduction rewriting scheme consisting of *XOR-Rewriting* and *Common-Rewriting* is proposed which helps to reduce the number of monomials by partially reducing the Gröbner basis. Furthermore several specific monomials are eliminated which fulfill a certain *Vanishing Constraint*.

The technique *XOR-Rewriting* of [30] eliminates all variables of the Gröbner basis which are neither an input nor an output of an XOR-gate. Also the primary input and output variables of the circuit are not eliminated in the Gröbner basis.

In our setting circuits are usually given as AIGs, hence we adopt this rewriting for AIGs by matching XOR (XNOR) patterns in the AIG which represent an XOR (XNOR) gate. This means we want to find a set of nodes for which the relation $s = \overline{(a \wedge b)} \wedge \overline{(\bar{a} \wedge \bar{b})}$ holds. We eliminate internal variables of these structures and define the polynomial of the XOR (XNOR) output directly in terms of the grandchildren.

### Example 4

The middle AIG in Fig. 1 depicts an XOR constraint. For this structure we only use the polynomial $-\,s+a+b-2ab$ for describing the logical constraint instead of the polynomials $-\,l+ab, -r + (1-a)(1-b)$, and $-\,s + (1-l)(1-r)$. This deletes polynomials containing the variables *l*, *r* from the Gröbner basis, unless they are used as an input of further gates.

After applying XOR-Rewriting the *Common-Rewriting* [30] technique further simplifies the Gröbner basis by eliminating all variables which are used exactly once as an input of a further gate. This technique can be compared to bounded variable elimination in SAT [18] after encoding a circuit to a CNF using, e.g., Tseitin encoding. This approach would also eliminate all variables in the CNF representing gates in the circuit having only one parent [20].

### Example 5

The right AIG of Fig. 1 contains several variables occurring only once, hence Common-Rewriting eliminates gates *t*, *u*, *v*, and *w*. Thus the relation of *r* is directly expressed in terms of *a*, *b*, *c*.

Although the concepts of XOR-Rewriting and Common-Rewriting seem rather intuitive in the sense that we can simply rewrite and delete polynomials from the Gröbner basis, we need sophisticated algebraic reasoning, i.e., elimination theory of Gröbner bases. We will introduce this theory in Sect. 5, but before doing so we want to complete the discussion of possible optimizations.

A further optimization presented in [30] was to add vanishing constraints, i.e., polynomials which are PCCs of the circuit *C* and because they are contained in *I*(*C*), they can be added to the Gröbner basis. In [30] a specific constraint was called the *XOR-AND Vanishing Rule*, denoting that an XOR-gate and AND-gate which have the same input can never be 1 at the same time. An XOR- and AND-gate with the same inputs logically represent a half-adder, where the XOR-gate represents the sum output and the AND-gate represents the carry output. Because a half-adder only sums up two bits, it can never happen that the sum output and carry output is 1 at the same time.

### Example 6

In the middle AIG of Fig. 1 the variable *l* represents an AND-gate and *s* represents an XOR-gate. Both have *a*, *b* as input. Hence we can deduce $s l = 0$.

We adapt this rule by searching for (negative) children or grand-children of specific AND-gates in the circuit. We add a corresponding polynomial to our Gröbner basis which deletes redundant monomials in intermediate reduction results.

Additionally to the above optimizations which we more or less adopted of [30], we presented in [8, 29] a further optimization called *Adder-Rewriting*, which is also based on elimination theory of Gröbner basis. The core idea is to simplify the Gröbner basis by introducing linear adder specifications.

### Definition 7

A sub-circuit $C_S$ of a circuit *C* is a *full-adder* if

$$\begin{aligned} -2 c - s + a + b + i \quad \text{ is } \text{ a } \text{ PCC } \text{ for } C \end{aligned}$$

for outputs *c*, *s* and inputs *a*, *b*, *i* of $C_S$ and a *half-adder* if

$$\begin{aligned} -2 c - s + a + b \quad \text{ is } \text{ a } \text{ PCC } \text{ for } C. \end{aligned}$$

We search for such sub-circuits representing full- and half-adders in the gate-level circuit *C*. Then we eliminate the internal variables of these sub-circuits, cf. Sect. 5, which has the effect that the linear adder specifications are included in the Gröbner basis. Reducing by these linear polynomials leads to substantial improvements in terms of computation time. Furthermore we will also add a polynomial representing the relation of *s* to the inputs *a*, *b*, *i*, because there are no restrictions on *s*. It can be used multiple times as a child of a gate and hence we need a relation for it. In general, assuming that the carry output *c* is always larger than the sum output *s*, the intermediate reduction polynomials includes the term $2c+s$ before we reduce *c*. Using the adder specification *s* is canceled in parallel during the reduction of *c*. Hence in certain multiplier architectures which consist only of full- and half-adders we never have to reduce *s*, cf. Sect. 10. But we have to include polynomials with leading term *s*, otherwise we lose completeness of our approach.

In [36] a similar strategy is given which detects embedded MAJ3 and XOR3 gates. In this approach the Gröbner basis of the circuit is not simplified, but the MAJ3 and XOR3 gates are used to receive a more efficient reduction order.

### Example 7

The middle AIG in Fig. 1 shows a half adder with outputs *l* and *s* as carry and sum and inputs *a*, *b*. Hence we can derive the relations $-2l-s+a+b$ and $-s+a+b-2ab$. In Fig. 2 the filled gates describe a full-adder. In this case we can obtain the specification $-2c_2 -s_2 + p_{20} + p_{11} +c_1$ by elimination of $g_0, g_1, g_2$.

We apply the optimizations in the following order: Adder-Rewriting, XOR-Rewriting, Common-Rewriting, Adding Vanishing Constraints. We start by eliminating variables from bigger parts of the circuit and continue with rewriting smaller parts and only in the end we add polynomials to the Gröbner basis.

In [29] we introduced a rewriting method which is different from the optimizations above, because in *Partial Product Elimination* we change the circuit specification. In multipliers where a partial product is simply the conjunction of two input bits, we find exactly $n^2$ polynomials, representing the corresponding AND-gates.

We can eliminate these polynomials by cutting off these gates from the circuit and verify them separately, e.g., we search for them in the AIG, but do not introduce separate polynomials $p_{i,j}=a_ib_j$. Hence we change the specification of the multiplier from Definition 5 to the specification given in Corollary 2.

### Corollary 2

A circuit *C* is a multiplier if

$$\begin{aligned} \sum _{i=0}^{2n-1} 2^i s_i - \sum _{i,j=0}^{n-1} 2^{i+j} p_{i,j} ~\in ~ I(C) \quad \text {with } ~p_{i,j}=a_ib_j. \end{aligned}$$

We can easily check that the specifications of Corollary 2 and Definition 5 are equivalent, when we expand the sums and replace every occurring of $p_{i,j}$ with $a_ib_j$ in Corollary 2.

This approach works only in multipliers with a simple partial product generation, in multipliers using, e.g., Booth encoding [26] these patterns do not exist, but it might be possible to find similar patterns in this situation too.

In the following we show how rewriting techniques, which are based on variable elimination can be applied to circuit verification.

## Variable elimination

Section 4 actually relies on elimination theory of Gröbner bases to justify our rewriting techniques. This section provides more details about this theory and also presents a theorem which allows to rewrite only local parts of the Gröbner basis following [8]. To apply these rewriting techniques the circuit is split into two parts by extracting a sub-circuit, which is then rewritten, without changing the rest of the circuit. For example Adder-Rewriting is applied on an extracted full- or half-adder and XOR-Rewriting is used for nodes in the AIG describing an XOR-constraint. Consequently also the overall ideal *I*(*C*) and the Gröbner basis *G* are split into two parts. In the extracted sub-circuit we want to eliminate redundant internal variables, i.e., variables occurring only inside the sub-circuit. For this purpose we use the elimination theory of Gröbner bases [16].

Recall, that if $I\subseteq \mathbb {Q}[X]$ and $J\subseteq \mathbb {Q}[X]$ are ideals, then their sum is the set $I+J = \{f+g \mid f \in I, g \in J \}$, which in fact is also an ideal in $\mathbb {Q}[X]$.

### Lemma 7

Let $I=\langle f_1, \ldots , f_r \rangle$ and $J=\langle g_1, \ldots , g_s \rangle$ be two ideals in $\mathbb {Q}[X]$. Then $I+J = \langle f_1, \ldots , f_r, g_1, \ldots , g_s \rangle$. In particular $\langle f_1, \ldots , f_r\rangle =\langle f_1 \rangle + \ldots + \langle f_r\rangle .$

### Proof

Property 2 and Corollary 3 in Chap. 4 §3 of [16].$\square$

In the simple case where all occurring polynomials are linear, the effect of elimination theory can be easily illustrated with Gaussian elimination.

### Example 8

(Gaussian elimination) Let us consider the following system of three linear equations in $\mathbb {Q}[x,y,z]$:

$$\begin{aligned} \begin{aligned} 2x+4y-3z+4=0 \\ 3x+7y-3z+2 =0 \\ 2x+5y-4z+5=0 \end{aligned} \end{aligned}$$

Let *V* be the vector space consisting of all $\mathbb {Q}$-linear combinations of the polynomials on the left hand side, then each possible root $(x,y,z)\in \mathbb {Q}^3$ of the above system is also a root of each polynomial contained in *V*. In this sense, *V* contains all linear polynomials whose solutions can be deduced from the roots of the system, i.e., the polynomials generating *V*.

If we are only interested in polynomials of *V* in which the variable *x* does not occur, we can triangularize the above system using Gaussian elimination. This for example leads to the equivalent system:

$$\begin{aligned} \begin{aligned} x + 2y - 2z + 3 = 0 \\ y + 3z - 7 = 0 \\ z - 2 = 0 \end{aligned} \end{aligned}$$

In Gaussian elimination new polynomials are derived by applying linear combinations of the original polynomials. Hence the polynomials on the left hand side belong to the vector space *V*. We see that two polynomials do not contain *x*. In fact, every element of *V* which does not contain *x* can be written as a linear combination of the polynomials $y+3z+2$ and $z+1$ which are free of *x*.

Since Gaussian elimination is defined only for linear equations we cannot use it for our setting, but using Gröbner bases theory we can extend the reasoning in the example above to systems of nonlinear equations.

In linear polynomials a term consists of a single variable, hence for triangularization we only have to order the terms in such a way that the variables which we want to eliminate are the largest terms. This ordering is generalized to multivariate terms by introducing an elimination order on the set of terms. In the following assume that we want to eliminate the variables belonging to a subset *Z* of *X*.

### Definition 8

[16] Let $X=Y{\mathop {\cup }\limits ^{\cdot }}Z$. An order < on the set of terms of [*X*] is called *elimination order* for *Z* if it holds for all terms $\sigma ,\tau$ where a variable from *Z* is contained in $\sigma$ but not in $\tau$, we obtain $\tau <\sigma$. We denote this ordering by $Y < Z$.

In the case that $Z=\{x_1,\dots ,x_i\}$ and $Y=\{x_{i+1},\dots ,x_n\}$, the lexicographic term order is such an elimination order. In Example 8 the elimination order $Y<Z$ is defined by a lexicographic ordering with $Y=\{y,z\}$ and $Z=\{x\}$.

### Definition 9

[16]  Assume an ideal $I\subseteq \mathbb {Q}[X]=\mathbb {Q}[Y,Z]$. The ideal where the *Z*-variables are eliminated is the *elimination ideal* $J \subseteq \mathbb {Q}[Y]$ defined by

$$\begin{aligned} J = I \cap \mathbb {Q}[Y]. \end{aligned}$$

### Theorem 3

[16] Given an ideal $I\subseteq \mathbb {Q}[X]=\mathbb {Q}[Y,Z]$. Further let *G* be a Gröbner basis of *I* with respect to an elimination order $Y < Z$. Then the set

$$\begin{aligned} H = G \cap \mathbb {Q}[Y] \end{aligned}$$

is a Gröbner basis of the elimination ideal $J = I \cap \mathbb {Q}[Y]$, in particular $\langle H \rangle = J$.

The requirements of Theorem 3 demand that we need to calculate a new Gröbner basis *H* w.r.t. to an elimination order $Y<Z$ for our circuit *C*. In general this means that we really need to apply Buchberger’s algorithm and cannot simply rely on the product criterion anymore as we did for *G*. Since Buchberger’s algorithm is computationally expensive [16], this is practically infeasible. In [8, 29] we derived a method which allows that we split *G* into two smaller Gröbner basis $G_A$ and $G_B$, where $\langle G_B \rangle$ defines the ideal generated by the gate polynomials of the extracted sub-circuit. The following theorem shows that in order to compute a basis of the elimination ideal $J=\langle G \rangle \cap \mathbb {Q}[Y]$ it suffices to compute a basis of the elimination ideal $\langle G_B\rangle \cap \mathbb {Q}[Y]$.

### Theorem 4

Let $G\subseteq \mathbb {Q}[X]=\mathbb {Q}[Y,Z]$ be a Gröbner basis with respect to some term order <. Let $G_A=G\cap \mathbb {Q}[Y]$ and $G_B=G{\setminus } G_A$. Let $<_Z$ be an elimination order for *Z* which agrees with < for all terms that are free of *Z*, i.e., terms free of *Z* are equally ordered in < and $<_Z$. Suppose that $\langle G_B\rangle$ has a Gröbner basis $H_B$ with respect to $<_Z$ which is such that every leading term in $H_B$ is free of *Z* or free of *Y*. Let $H_B = H_Y \cup H_Z$, such that $H_Z$ consists of all polynomials with leading terms in *Z* and $H_Y = H_B {\setminus } H_Z$ contains the remaining polynomials with leading terms in *Y*. Then

1. $$\langle G \rangle \cap \mathbb {Q}[Y] = (\langle G_A\rangle +\langle G_B\rangle )\cap \mathbb {Q}[Y]=\langle G_A\rangle +(\langle G_B\rangle \cap \mathbb {Q}[Y])$$.
2. $H = G_A \cup H_Y$ is a Gröbner basis for $\langle G_A\rangle +(\langle G_B\rangle \cap \mathbb {Q}[Y])$ w.r.t. the ordering $<_Z$.

### Proof

**Fig. 3:**
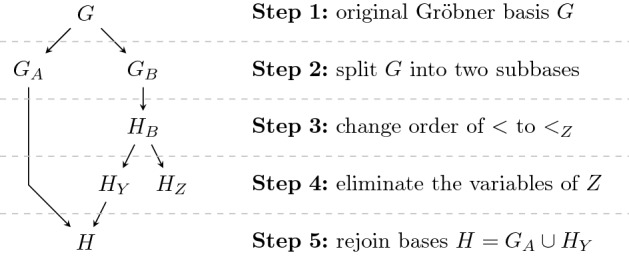
Each step of the elimination procedure of the proof of Theorem 4

(1) The steps of the elimination process of this proof are depicted in Fig. 3. Since $Y<_ZZ$, it follows that the polynomials in $H_Y$ cannot contain any variable of *Z*. Furthermore by definition $G_A$ does not contain any polynomial containing *Z*-variables, hence variables of *Z* only occur in $H_Z$.

By Lemma 7 we derive

$$\begin{aligned} \begin{aligned} \langle G \rangle&= \langle G_A \rangle + \langle G_B \rangle =\langle G_A \rangle + \langle H_B \rangle \\&=\langle G_A \rangle + \langle H_Y \rangle + \langle H_Z \rangle =\langle G_A \cup H_Y \rangle + \langle H_Z \rangle . \end{aligned} \end{aligned}$$

By $$ GB (S,o)$$ we denote an arbitrary Gröbner basis for *S* w.r.t. an ordering *o*. Changing an arbitrary basis into a Gröbner basis does not affect the ideal, hence

$$\begin{aligned} \begin{aligned} \langle G_A \cup H_Y \rangle + \langle H_Z \rangle&=\langle GB (G_A \cup H_Y,<_Z) \rangle + \langle H_Z \rangle \\&=\langle GB (G_A \cup H_Y, <_Z) \cup H_Z \rangle . \end{aligned} \end{aligned}$$

Furthermore $GB (G_A \cup H_Y, <_Z) \cup H_Z$ is a Gröbner basis, because all S-polynomials of pairs of polynomials *p*, *q* reduce to zero:

1. $p,q \in GB (G_A \cup H_Y, <_Z)$: By Lemma 2, ${\text {spol}}(p,q)$ reduces to zero.
2. $p \in GB (G_A \cup H_Y, <_Z), q \in H_Z$: The leading terms of $H_Z$ contain only variables of *Z*, whereas the polynomials $G_A \cup H_Y$ do not contain any variable of *Z*. Hence by Lemma 3, ${\text {spol}}(p,q)$ reduces to zero.
3. $p,q \in H_Z$: Since $H_B = H_Y \cup H_Z$ is a Gröbner basis, it holds that ${\text {spol}}(p,q)$ reduces to zero w.r.t. $H_B$. Consequently it reduces to zero w.r.t. $G_A \cup H_B = G_A \cup H_Y \cup H_Z$. Since each leading term of $G_A\cup H_Y$ is a multiple of a leading term in $GB (G_A\cup H_Y,<_Z)$, ${\text {spol}}(p,q)$ reduces to zero w.r.t. $GB (G_A\cup H_Y,<_Z) \cup H_Z$.

Combining the above results we conclude that $GB (G_A \cup H_Y, <_Z) \cup H_Z$ is a Gröbner basis for the ideal $\langle GB (G_A \cup H_Y, <_Z) \cup H_Z\rangle =\langle G \rangle$. Following Theorem 3 we receive

$$\begin{aligned} \begin{aligned}&(\langle G_A \rangle + \langle G_B \rangle ) \cap \mathbb {Q}[Y] \\&\quad ={}\langle GB (G_A \cup H_Y,<_Z) \cup H_Z \rangle \cap \mathbb {Q}[Y] \\&\quad ={}\langle GB (G_A \cup H_Y, <_Z) \rangle . \end{aligned} \end{aligned}$$

Since computation of a Gröbner basis does not change the ideal, we have

$$\begin{aligned} \langle GB (G_A \cup H_Y, <_Z) \rangle =\langle G_A \cup H_Y \rangle =\langle G_A \rangle + \langle H_Y \rangle . \end{aligned}$$

Because the set $H_Y$ does not contain any variable of *Z*, it follows

$$\begin{aligned} \begin{aligned} \langle H_Y \rangle =\langle H_B \rangle \cap \mathbb {Q}[Y] =\langle G_B \rangle \cap \mathbb {Q}[Y] \end{aligned} \end{aligned}$$

Altogether by composing the derived results we obtain

$$\begin{aligned} \begin{aligned} (\langle G_A \rangle + \langle G_B \rangle ) \cap \mathbb {Q}[Y] = \langle G_A \rangle + (\langle G_B \rangle \cap \mathbb {Q}[Y]). \end{aligned} \end{aligned}$$

(2) We need to prove that for every term $\tau \in [Y]$ which is also a leading term of a polynomial in $\langle G \rangle$ it follows that there exists a polynomial $f \in G_A \cup H_Y$ such that ${\text {lt}}(f)\mid \tau$. Let $\tau$ be such a term.

Because *G* is a Gröbner basis it holds that there exists a $g \in G$ with ${\text {lt}}(g)\mid \tau$. Since $G = G_A \cup G_B$ it consequently follows that either $g \in G_A$ or $g \in G_B$:

1. $g \in G_A$: It immediately follows that $g \in G_A \cup H_Y$, hence $f:=g$.
2. $g \in G_B$: Since $\langle G_B \rangle = \langle H_B \rangle$, there exists an element $h \in H_B$ with ${\text {lt}}(h)\mid {\text {lt}}(g)$. From this it follows that ${\text {lt}}(h)\mid \tau$. Since $\tau \in [Y]$ it further holds that ${\text {lt}}(h)\in [Y]$. Hence $h \in H_Y$ and altogether $h \in G_A \cup H_Y$. In this case $f:=h$.

So for each $g \in G$ we find $f \in G_A \cup H_Y$ whose leading term divides $\tau$. $\square$

The above theorem allows that we simply add the Gröbner basis $H_Y$ of the elimination ideal of the extracted sub-circuit $\langle H_Y \rangle = \langle H_B \rangle \cap \mathbb {Q}[Y] = \langle G_B \rangle \cap \mathbb {Q}[Y]$ to the Gröbner basis $G_A$ of the remaining circuit and obtain again a Gröbner basis, preventing that we have to compute a new Gröbner basis w.r.t. to an elimination order for the whole circuit *C*. Actually we only have to really compute one “small” local Gröbner basis $H_B$. Although in principle we can choose *Z* arbitrarily, we apply the idea to sets of variables that only occur locally in the circuit. One source of such variables are intermediate results of adders.

### Example 9

We want to apply Adder-Rewriting on the full adder in the circuit *C* of Fig. 2, which is depicted by the colored gates. The full adder has outputs $c_2$ (carry) and $s_2$ (sum) and three inputs $p_{20}, p_{11}, c_1$. The internal gates $g_2, g_1, g_0$ are not used outside the full adder structure, hence we want to eliminate them and include the specification $2c_2+s_2 -p_{20}- p_{11}- c_1$ in the Gröbner basis *G*.

The Gröbner basis *G* which is depicted by polynomials in the right column in Fig. 2 is split such that $G_A=G{\setminus } G_B$ and

$$\begin{aligned} G_B= & {} ~\{-g_0 + p_{20} + p_{11} - 2p_{20}p_{11},\quad -g_1 + p_{20}p_{11}, \quad -g_2 + c_1g_0, \\&\quad -s_2 + c_1 + g_0 - 2c_1g_0, \quad -c_2 + g_1 + g_2 - g_1g_2\} \end{aligned}$$

We apply variable elimination only in $G_B$. For this let $Z=\{g_2, g_1, g_0\}$. According to the requirements of Theorem 3 we need to find an elimination order $<_Z$ such that $Y<Z$. So far we used in Example 3 a lexicographic term ordering < with

$$\begin{aligned}\begin{aligned}&b_0< b_1< a_0< a_1< a_2< p_{00}< s_0< p_{01}< p_{10}< s_1< c_1 \\&<p_{11}<p_{20}< {{g_0}}< {{g_1}}< {{g_2}}< s_2< c_2< p_{21}< s_3< c_3< s_4 \end{aligned} \end{aligned}$$

We choose $<_Z$ such that < and $<_Z$ restricted on *Y* are equal, i.e., we move $g_0,g_1,g_2$ to be the largest variables in the lexicographic ordering, but do not change the order of the remaining variables.

We compute a Gröbner basis $H_B$ w.r.t. $<_Z$. During the computation we use the notation $f \xrightarrow {P} r$, meaning that *r* is the remainder *f* with respect to *P*. For simplification we immediately reduce higher powers without showing this reduction by the field polynomials explicitly. Initially $H_B$ contains:

$$\begin{aligned} \begin{array}{lll} f_1:=-g_0 - 2p_{20}p_{11} + p_{20} + p_{11} ,&{}\quad f_2:= -g_1 + p_{20}p_{11}, &{}\quad f_3:= -g_2 + g_0c_1, \\ f_4:= - 2c_1g_0+ g_0-s_2 + c_1, &{}\quad f_5:= - g_2g_1 + g_2 + g_1 -c_2 &{} \end{array} \end{aligned}$$

According to Buchberger’s algorithm [11] we consider all possible pairs $(f_i, f_j) \in H_B \times H_B$ and compute ${\text {spol}}(f_i,f_j)\xrightarrow {H_B} r$. If *r* is not zero, we add *r* to $H_B$. This step is repeated until all ${\text {spol}}(f_i,f_j)$ for $(f_i, f_j) \in H_B \times H_B$ reduce to zero.

Initially we only have to explicitly compute the remainders of ${\text {spol}}(f_1,f_4)$, ${\text {spol}}(f_2,f_5)$ and ${\text {spol}}(f_3,f_5)$, because all other S-Polynomials reduce to zero according to the product criterion, cf. Lemma 3.

$$\begin{aligned} \begin{aligned} {\text {spol}}(f_1,f_4)&= ~2c_1f_1 - f_4 = -g_0+s_2 - 4p_{20}p_{11}c_1+2p_{20}c_1+2p_{11}c_1-c_1 \\&\xrightarrow {\{f_1\}} s_2-4p_{20}p_{11}c_1+2p_{20}p_{11}+ 2p_{20}c_1-p_{20}+ 2p_{11}c_1-p_{11}-c_1 =:f_6 \end{aligned} \end{aligned}$$

The non-zero remainder $f_6$ of ${\text {spol}}(f_1,f_4)$ is added to $H_B$. Since ${\text {lt}}(f_6)$ is coprime to all other leading terms of $H_B$, all ${\text {spol}}(f_i, f_6)$ reduce to zero, cf. Lemma 3.

$$\begin{aligned}\begin{aligned} {\text {spol}}(f_2,f_5)&= g_2f_2 - f_5 = g_2p_{20}p_{11}-g_2-g_1+c_2\\&\xrightarrow {\{f_3\}} -g_1 + g_0p_{20}p_{11}c_1 - g_0c_1 + c_2 \\&\xrightarrow {\{f_2\}} g_0p_{20}p_{11}c_1 - g_0c_1 + c_2 - p_{20}p_{11} \\&\xrightarrow {\{f_1\}} c_2 + 2 p_{20}p_{11}c_1 - p_{11}c_1 + p_{20}c_1 - p_{20}p_{11} =:f_7 \end{aligned} \end{aligned}$$

We add $f_7$ to $H_B$ and we again apply the product criterion for all S-Polynomials containing $f_7$.

$$\begin{aligned}\begin{aligned} {\text {spol}}(f_3,f_5) \quad =&~ g_1f_3 - f_5 = -g_2 + g_1g_0c_1-g_1+c_2 \\ \xrightarrow {\{f_3\}}&~ g_1g_0c_1-g_1-g_0c_1+c_2 \\ \xrightarrow {\{f_2\}}&~ g_0p_{20}p_{11}c_1 - g_0c_1 + c_2 - p_{20}p_{11} \\ \xrightarrow {\{f_1\}}&~ c_2 + 2 p_{20}p_{11}c_1 - p_{11}c_1 + p_{20}c_1 - p_{20}p_{11}\xrightarrow {\{f_7\}}0 \end{aligned} \end{aligned}$$

At this point the algorithm terminates, because now all S-Polynomials reduce to zero. Thus $H_B = \{f_1, f_2, f_3, f_4, f_5, f_6, f_7\}$ is a Gröbner basis for $\langle H_B \rangle$.

Although $H_B$ is already a Gröbner basis, we will modify it to cover our needs. It is always allowed to add polynomials of $\langle H_B\rangle$ to $H_B$ without violating the Gröbner basis property. In order to add the specification of the full adder to $H_B$ we construct $f_8 := 2f_7+f_6 = 2c_2+s_2-p_{20}-p_{11}-c_1$ and add it to $H_B$.

To reduce the size of the Gröbner basis $H_B$ we eliminate unnecessary polynomials. Lemma 3 in Chap. 2 §7 of [16] tells us that we can remove a polynomial *p* from our Gröbner Basis $H_B$ whenever we have a further polynomial $q \in H_B$ such that ${\text {lt}}(q)\mid {\text {lt}}(p)$. Thus we can eliminate $f_4,f_5$ and $f_7$ and our final Gröbner basis $H_B$ w.r.t. $<_Z$ is

$$\begin{aligned} \begin{aligned} H_B = \{&{g_0+2p_{20}p_{11}-p_{20}-p_{11}},\quad {g_1-p_{20}p_{11}}, \quad {g_2+g_0c_1},\\&s_2-4p_{20}p_{11}c_1+2p_{20}p_{11}+ 2p_{20}c_1-p_{20}+ 2p_{11}c_1-p_{11}-c_1, \\&2c_2+s_2-p_{20}- p_{11}-c_1 \}. \end{aligned} \end{aligned}$$

We eliminate the first three colored polynomials containing variables of *Z* and derive $\langle H\rangle = \langle G_A \rangle + \langle H_Y \rangle$ with $H_Y =H_B \cap \mathbb {Q}[Y]$.

**Fig. 4:**
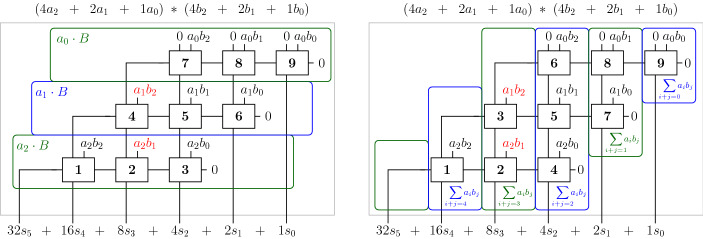
Standard row-wise slicing (left) and our column-wise slicing (right) for a clean 3-bit carry-save-adder based (CSA) multiplier. The numbers in the full-adders depict the order

## Order

As long as the gate and field polynomials are ordered according to a reverse topological lexicographic term order, the choice of order does not affect the correctness of the approach, cf. Theorem 1. However the choice of order has an influence on the number of monomials in the intermediate reduction result [35]. Hence, in addition to rewriting and reducing the Gröbner basis *G*, choosing an appropriate term and hence reduction order has a tremendous effect on computation time.

Given the two dimensional structure of multipliers, two orderings seem well fitted, namely a row-wise and a column-wise ordering. The idea in both approaches is to partition the gates of a circuit into *slices*, which are then totally ordered. The gates within a slice are ordered reverse topologically. The combined order of the variables has to be reverse topological, such that the requirements of Theorem 1 are fulfilled and hence the gate and input field polynomials form a Gröbner basis.

In the row-wise approach the gates are ordered according to their backward level. The ordering is abstractly depicted in the left circuit in Fig. 4, where the order of the full-adders in a clean carry-save-adder based (CSA) multiplier is given. Informally, a multiplier is *clean* when neither gate synthesis nor mapping is applied and where the XOR-gates, partial products and the half/full adders can easily be identified. Otherwise a multiplier is called *dirty*. In previous work the row-wise approach is widely used. In the approach of [35] the gates are ordered according on their logic level based on the circuit inputs. In [14] the row-wise order is used to derive a word-level specification for a CSA step in a clean CSA multiplier. Unfortunately, the variable order is only roughly discussed in [30].

In the column-wise order, cf. right side of Fig. 4, the multiplier is partitioned vertically such that each slice contains exactly one output bit. We will use this order to determine a more robust incremental checking approach.

In Fig. 4 we also list the sum of the partial products which occur in the row-wise and column-wise slices. Assume we swap $a_1b_2$ and $a_2b_1$. In contrast to permuting partial products within a row, permuting partial products within a column does not affect the correctness of the multiplier. By exchanging $a_1b_2$ and $a_2b_1$ the given sums of partial products for the row-wise slices are not valid anymore, whereas in the column-wise slicing the sum of partial products is still correct, meaning we can uniquely identify the partial products in a column-wise slice.

## Incremental column-wise checking

The goal of an incremental checking algorithm is to divide the verification problem into smaller, less complex and thus more manageable sub-problems. Because a column-wise term order is robust under permutation of partial products, we use such an order to define our incremental slices. Furthermore we split the word-level specification of Definition 5 into smaller specifications which relate the partial products, incoming carries, sum output bit and the outgoing carries of each slice.

### Definition 10

Let *C* be a circuit which is partitioned according to a column-wise term order, such that each slice contains exactly one output bit. For column *i* with $0 \le i < 2n$ let $P_i = \sum _{k+l = i} a_kb_l$ be the *partial product sum* (of column *i*).

### Definition 11

Let *C* be a circuit, as defined in Sect. 3. A sequence of $2n+1$ polynomials $C_0, \ldots , C_{2n}$ over the variables of *C* is called a *carry sequence* if

$$\begin{aligned} -\,C_i + 2C_{i+1}+s_i-P_i \in I(C) \quad \text{ for } \text{ all } \,\,0 \le i < 2n+1 \end{aligned}$$

Then the $R_i = -\,C_i + 2C_{i+1}+s_i-P_i$ polynomials are called the *carry recurrence relations* for the sequence $C_0, \ldots , C_{2n}$.

Based on these definitions we can obtain a general theorem which allows to incrementally verify multiplier circuits using carry recurrence relations. For this theorem it is not necessary to know how the carry sequence is actually derived.

### Theorem 5

Let *C* be a circuit where all carry recurrence relations are contained in *I*(*C*), i.e., $C_0, \ldots , C_{2n}$ define a carry sequence as in Definition 11. Then *C* is a multiplier in the sense of Definition 5, if and only if $C_0 - 2^{2n}C_{2n} \in I(C)$.

### Proof

By the condition of Definition 11, we have (modulo *I*(*C*))

$$\begin{aligned} \sum _{i=0}^{2n-1}2^is_i&= \sum _{i=0}^{2n-1}2^i \left( P_i + C_{i}-2C_{i+1}\right) \\&= \sum _{i=0}^{2n-1} 2^i P_i + \underbrace{\sum _{i=0}^{2n-1} \left( 2^i C_i - 2^{i+1} C_{i+1}\right) }_{\displaystyle C_0 - 2^{2n}C_{2n}}. \end{aligned}$$

It remains to show $\sum _{i=0}^{2n-1}2^iP_i = \left( \sum _{i=0}^{n-1}2^ia_i\right) \left( \sum _{i=0}^{n-1}2^ib_i\right)$:

$$\begin{aligned} \begin{aligned} \sum _{i=0}^{2n-1}2^iP_i = \sum _{i=0}^{2n-1}2^i \sum _{\begin{array}{c} k+l=i\\ k,l \ge 0 \end{array}}^{k,l \le n-1}a_kb_l = \sum _{k=0}^{n-1}\sum _{l=0}^{n-1}2^{k+l}a_kb_l = \left( \sum _{k=0}^{n-1}2^{k}a_k \right) \left( \sum _{l=0}^{n-1}2^{l}b_l\right) \end{aligned} \end{aligned}$$

Putting the above calculations together yields:

$$\begin{aligned} \underbrace{\sum \limits _{i=0}^{2n-1}2^is_i}_L \;=\; \underbrace{\left( C_0 - 2^{2n}C_{2n}\right) }_{L_1} \,+\, \underbrace{\left( \sum \limits _{k=0}^{n-1}2^{k}a_k\right) \left( \sum \limits _{l=0}^{n-1}2^{l}b_l\right) }_{L_2} \end{aligned}$$

Since all $R_i \in I(C)$, it holds that $L-L_1-L_2 \in I(C)$. For soundness, we assume $L_1 \in I(C)$, thus conclude $L - L_2 \in I(C)$, which proves that *C* is a multiplier. For completeness, let $L - L_2\in I(C)$ and thus $L_1 \in I(C)$. $\square$

**Fig. 5:**
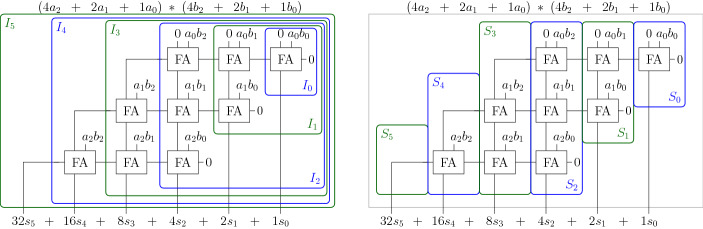
Deriving input cones (left) and slices (right) for a clean 3-bit CSA multiplier

For our incremental checking algorithm we determine for each output bit $s_i$ its input cone, namely the gates which $s_i$ depends on (cf. left side of Fig. 5):

$$\begin{aligned} I_{i} :=\{\text{ gate } g \mid g \text{ is } \text{ in } \text{ input } \text{ cone } \text{ of } \text{ output } s_i \} \end{aligned}$$

We derive slices $S_i$ as the difference of consecutive cones $I_i$ (cf. right side of Fig. 5):

$$\begin{aligned} S_0 := I_0 \qquad S_{i+1} :=I_{i+1}{\setminus } \bigcup _{j=0}^i S_j \end{aligned}$$

### Definition 12

(*Sliced Gröbner Bases*) Let $G_i$ be the set of polynomial representations of the gates in a slice $S_i$, cf. Eq. 1, and the input field polynomials. The terms are ordered such that the requirements of Theorem 1 are fulfilled.

### Corollary 3

The set $G_i$ is a Gröbner basis for the slice ideal $\langle G_i \rangle$.

### Proof

Follows from Theorem 1 with *C* replaced by $S_i$ and *G* replaced by $G_i$.$\square$

Since the ideal $\langle G_i \rangle$ contains all the field polynomials $F_i$ for the gate variables in $S_i$, we may use them in the reduction process to eliminate exponents greater than 1 in the intermediate reduction results. Our incremental checking algorithm, cf. Algorithm 2, works as follows: We start at the last output bit $s_{2n-1}$ and compute the polynomials $C_i$ recursively as the remainder of dividing $2C_{i+1}+s_i-P_i$ by $G_i \cup F_i$. Hence a polynomial $C_i$ is uniquely defined, given $P_i$ and $C_{i+1}$. It remains to fix the boundary polynomial $C_{2n}$, where we simply choose $C_{2n} = 0$.

### Theorem 6

Algorithm 2 returns $true$ iff *C* is a multiplier.

### Proof

By definition $R_i := -C_i + 2C_{i+1}+s_i-P_i \in \langle G_i \cup F_i\rangle$. Let *F* denote the set of all field polynomials for the variables of *C*. Since $G_i \subseteq G$ and $F_i \subseteq F$, we have $G_i \cup F_i \subseteq G \cup F$. Furthermore $\langle G \cup F \rangle = \langle G \rangle = J(C)$ and thus $R_i \in J(C) = I(C)$.

We show inductively that $C_i$ is reduced w.r.t. $U_i$, where $U_i := \bigcup _{j\ge i} (G_j \cup F_j)$. This requires that $s_i$ and $P_i$ are reduced w.r.t. to $U_{i+1}$, which holds due to the construction of the sliced Gröbner bases $G_i$. By $U_0 = G \cup F$ we can derive that the final remainder $C_0$ is reduced w.r.t. $G \cup F$ thus $C_0 = C_0 - 2^{2n}C_{2n} \in I(C) = J (C)$ iff $C_0 = 0$, which concludes the proof using Theorem 5.$\square$

Consequently Algorithm 2 returns $false$ iff a multiplier is incorrect, i.e., $C_0 \ne 0$. As discussed in the final remark of Sect. 3 we can use $C_0$ to receive a concrete counter-example. It also is possible to abort the algorithm earlier if we find partial products $a_kb_l$ of higher slices $S_{k+l} = S_j$ in remainders $C_i$ with $i < j$.
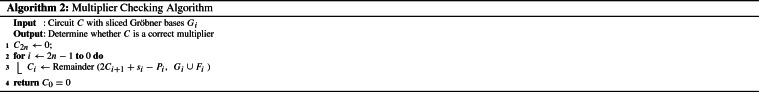


## Incremental equivalence checking

In this section we introduce an incremental equivalence checking algorithm [29] generalizing our incremental checking approach to gate-level equivalence checking of two multipliers, but the approach is not restricted to multiplier circuits only. The presented theory applies to all acyclic circuits $C, C'$ which have the same inputs and the same number of output bits. We generalize our definition of circuits of Sect. 3 as follows.

Let *C* be a circuit with *l* boolean inputs $a_0, \ldots ,a_{l-1}$ and *m* outputs $s_0, \ldots , s_{m-1}$. Internal gates are represented by $g_0, \ldots , g_j$. Further let $C'$ be a circuit with the same *l* boolean inputs, but *m* different outputs $s_0', \ldots , s_{m-1}'$. The gates of $C'$ are defined by gate variables $g_0', \ldots , g_k'$. The union of $C, C'$ is denoted by $C \cup C'$, for which we can determine $I(C\cup C')=J(C\cup C')$ as described in Sect. 3.

The core idea of equivalence checking is to verify that two circuits *C* and $C'$ compute the same output, given the same input. The benefit of equivalence checking is that a circuit can be verified without requiring a word-level specification by checking the equivalence of a circuit and a correct “golden” reference circuit. In the following we show how we can derive an incremental equivalence checking approach based on our column-wise checking algorithm of Sect. 7.

### Definition 13

Let $C, C'$ be two circuits. They are *equivalent*, written $C \equiv C'$, if

$$\begin{aligned} s_i - s_i' \qquad \in I(C \cup C')\quad i = 0,\ldots ,m-1. \end{aligned}$$

### Lemma 8

$$\begin{aligned} C \equiv C' \quad \text{ iff }\quad \sum _{i=0}^{m-1}2^i\left( s_i - s_i'\right) \in I\left( C \cup C'\right) \end{aligned}$$

### Proof

“$\Rightarrow$”: Follows from Definition 2.

“$\Leftarrow$”: Let $\varphi :X \rightarrow \mathbb {B} \subseteq \mathbb {Q}$ denote an evaluation of all variables *X* of $C, C'$, which is implied by the functionality of the circuit gates, e.g., values of $s_i, s_i'$ in $\mathbb {B}$ are uniquely determined given fixed inputs $a_0, \ldots , a_{l-1}$. We extend $\varphi$ to an evaluation of polynomials in the natural way (the unique homomorphic extension), i.e., $\varphi :\mathbb {Q}[X] \rightarrow \mathbb {Q}$. For all PCCs *f*, i.e. $f \in I(C \cup C')$, it holds by definition that $\varphi (f) = 0$. Since $\varphi (s_i), \varphi (s_i') \in \mathbb {B}$ it is clear that $\varphi (s_i - s_i') \in \{-1,0,1\}$.

Assume $\sum _{i=0}^{m-1}2^i(s_i - s_i') \in I(C \cup C')$, but $C \not \equiv C'$. Then there is a largest *k* with $0\le k < m$ and $\varphi (s_k - s_k') \ne 0$, which gives the following contradiction

$$\begin{aligned} 0= & {} \displaystyle \varphi \left( \sum _{i=0}^{m-1}2^i(s_i - s_i')\right) ~=~ \sum _{i=0}^{k}2^i\varphi \left( s_i - s_i'\right) \\= & {} \underbrace{2^k\varphi \left( s_k - s_k'\right) }_{{} \in \{-2^k,2^k\}} \,+\, \underbrace{\displaystyle \sum _{i=0}^{k-1}2^i\varphi \left( s_i - s_i'\right) }_{{} \in [-2^k+1, 2^k-1]} ~\ne ~ 0 \end{aligned}$$

$$\square $$

As for the incremental checking algorithm we define a sequence of relations, which is used to split the word-level equivalence specification. Based on the sequence we define an abstract incremental bit-level equivalence checking algorithm.

### Definition 14

Let $C, C'$ be two circuits. A sequence of *m* polynomials $\Delta _0, \ldots , \Delta _{m}$ over the variables of *C*, $C'$ is called a sequence of *slice polynomials* if

$$\begin{aligned} - \Delta _i + 2 \Delta _{i+1} + \left( s_i - s_i'\right) \in I\left( C \cup C'\right) \quad \text{ for } \text{ all } \,\,0 \le i < m \end{aligned}$$

The polynomials $E_i = - \Delta _i + 2 \Delta _{i+1} + (s_i - s_i')$ are called *slice relations* for the sequence $\Delta _0, \ldots , \Delta _{m}$.

### Theorem 7

Let $C, C'$ be two circuits and $\Delta _0, \ldots , \Delta _{m}$ be a sequence of slice polynomials. Then $C \equiv C'$ in the sense of Definition 13 iff $2^{m}\Delta _{m} - \Delta _0 \,\in \, I(C \cup C')$.

### Proof

Using Definition 14 we obtain modulo $I(C \cup C')$

$$\begin{aligned} \sum _{i=0}^{m-1}2^i(s_i - s_i') = {\sum _{i=0}^{m-1} 2^i (2 \Delta _{i+1} - \Delta _i)}={\displaystyle 2^{m}\Delta _{m} - \Delta _0 }. \end{aligned}$$

$$\square $$

Before we can define our incremental equivalence checking algorithm, we need to find a Gröbner basis for the ideal $I(C \cup C')$ and similar to Sect. 7 we will define input cones which are then used to define slices $S_i$.

### Lemma 9

Let *C* and $C'$ be two circuits. Let $G, G'$ be Gröbner bases for $I(C), I(C')$ w.r.t. $\le , \le '$, satisfying the conditions of Theorem 1. Further let $\le _\cup$ be such that $\le , \le '$ are contained in $\le _\cup$. Then $G \cup G'$ is a Gröbner basis for $I(C \cup C')$ w.r.t. $\le _\cup$.

### Proof

The set $G \cup G'$ consists of all gate polynomials of $C, C'$ and input field polynomials $a_i(a_i-1)$, but no more. Since all variables of $C, C'$ apart from the input variables are unequal, $G \cap G'$ contains only the input field polynomials.

Since the variables $a_0, \ldots ,a_{l-1}$ are the smallest elements in $\le , \le '$ they are by definition also the smallest elements in $\le _\cup$. Furthermore the term orderings for the gate polynomials of *C* and $C'$ are still valid in $\le _\cup$. Hence by the constraints on $\le _\cup$ the leading term of a polynomial in $G \cup G'$ is either the output variable of a circuit gate or the square of an input variable. Thus by Lemma 3$G \cup G'$ is a Gröbner basis for $I(C \cup C')$ w.r.t. $\le _\cup$.$\square$

For each pair of output bits $s_i$ and $s_i'$ we determine its input cone

$$\begin{aligned} I_{i} \,:=\,\left\{ \text{ gate } g \mid g \text{ is } \text{ in } \text{ input } \text{ cone } \text{ of } \text{ output } s_i \text{ or } s_i' \right\} . \end{aligned}$$

The slices $S_i$ are defined as in Sect. 7 as difference of consecutive cones $I_i$. For each slice we define a set of polynomials $G_i$ according to Definition 12. By Corollary 3 such a set is a Gröbner basis for the ideal generated by the input field polynomials and the gate polynomials of a slice. Note that the ideal generated by $G_i$ contains all the field polynomials $F_i$ for the gate variables in $S_i$.

Using Theorem 7 we define our incremental equivalence checking algorithm, cf. Alg 3. Setting the boundary $2^{m}\Delta _{m}$ to 0 we obtain the sequence of slice polynomials $\Delta _0,\ldots ,\Delta _{m-1}$ recursively by computing each $\Delta _i$ as the remainder of $2\Delta _{i+1}+s_i-s_i'$ modulo the sliced Gröbner bases $G_i \cup F_i$. This ensures that all $E_i$ are contained in $\langle G_i \cup F_i \rangle \subseteq I(C \cup C')$. After computing $\Delta _0,\ldots ,\Delta _{m-1}$ we have to check if $\Delta _0 = 0$.

By similar arguments as in the proof of Theorem 6 we show correctness of Algorithm 3.
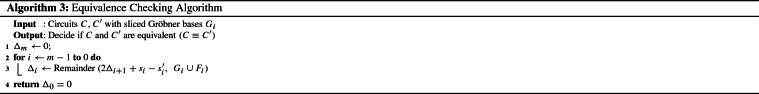


### Theorem 8

Algorithm 3 returns *true* iff $(C \equiv C')$.

### Proof

It holds by definition that $E_i= - \Delta _i + 2 \Delta _{i+1} + (s_i - s_i') \in \langle G_i \cup F_i \rangle$. By *F* we denote the set of all field polynomials of the variables of $C,C'$. We can derive that $G_i \cup F_i \subseteq G \cup G' \cup F$ Therefore $E_i \in \langle G \cup G' \cup F \rangle = I(C \cup C')$.

We show inductively that $\Delta _i$ is reduced w.r.t. $U_i := \bigcup _{j\ge i} (G_j \cup F_j)$. For the induction it is required that $s_i$ and $s_i'$ are reduced w.r.t. to $U_{i+1}$, which holds due to the definition of the sliced Gröbner bases. With $U_0 = G \cup G' \cup F$ we get $\Delta _0$ is reduced w.r.t. $G \cup G' \cup F$ thus $\Delta _0 = 2^{m}\Delta _{m}-\Delta _0 \in J (C \cup C')$ iff $\Delta _0 = 0$, concluding the proof using Theorem 7. $\square$

## Engineering


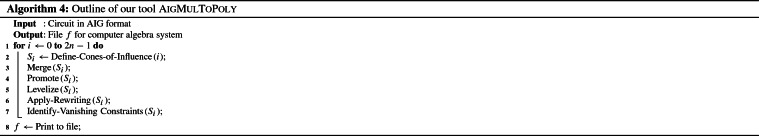


In this section we present the outline of our tool AigMulToPoly [28], cf. Algorithm 4, and present a novel approach to define column-wise slices. Our tool AigMulToPoly, which is implemented in *C*, takes a circuit given as an AIG in AIGER format [7] as input and returns a file which can be passed on to the computer algebra systems Mathematica [34] and Singular [17].

In AigMulToPoly we define the cones-of-influence, which are used to define the column-wise slices. In certain cases we have to optimize the slices by moving nodes from one slice to another slice, which we discuss further down. After slicing an ordering is defined for the nodes inside a slice, the rewriting methods are applied and as a last step everything including the computation instructions of our incremental column-wise verification algorithm in the syntax of the computer algebra system is printed to a file. In the computer algebra system the actual computation (repeated multivariate division) of the incremental checking algorithm is executed.

We generally define the column-wise slices based on the input cones, cf. Sect. 7. But this is not always precise enough for dirty multipliers. It frequently happens, that AIG-nodes which are not directly used to compute the output $s_i$ of a slice are allocated to later slices. This happens for example for carry outputs of full- and half-adders when they do not share their nodes with the sum output.

### Example 10

In Fig. 2 the dashed lines depict an optimal column-wise slicing. If we would define the slices only based on input cones, then the AND-gate with output $c_1$ would belong to $S_2$. Similar for the gates with outputs $c_2, g_2, g_1, c_3$, thus all the full- and half-adders would be cut into two pieces.

We want to have these nodes in the same slice as the nodes computing the sum output of an adder. Otherwise we cannot apply Adder-Rewriting. We informally define those nodes in a slice $S_i$ which are used as inputs of nodes in a slice $S_j$ with $j>i$ as *carries of a slice* $S_i$. The size of the carry polynomial $C_i$ can be reduced by decreasing the number of carries of the corresponding slice $S_i$. If the nodes are not moved, larger carry polynomials $C_i$ are generated and hence we get larger intermediate reduction results than necessary. Therefore we eagerly move nodes between slices in a kind of peephole optimization, backward (*Merge*) as well as forward (*Promote*).

**Fig. 6:**
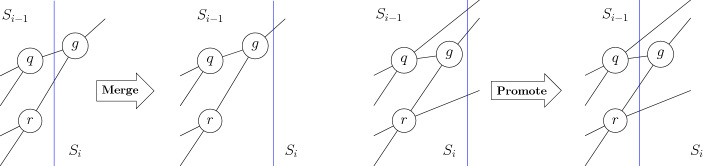
Moving nodes between slices by Merge (left side) and Promote (right side)

*Merge* Assume we find a node *g* in the AIG which belongs to a slice $S_i$ and both children *q* and *r* belong to smaller slices $S_j$ and $S_k$. Let $l=\text{ max }(j,k)$. If the children *q* and *r* do not have any other parent than *g* in a bigger slice than $S_l$, we move the node *g* back to slice $S_l$. This approach is depicted on the left side of Fig. 6 for $j=k=i-1$. Thus after merging *g*, the nodes *q*, *r* are less likely to be carry variables any more, especially when $j=k$. We apply merging repeatedly until completion and $S_{l}$ and $S_{i}$ are updated after each application. Merging nodes usually ensures that the complete logic of a full- or half-adder is contained within one slice.

### Example 11

In the circuit of Fig. 2 gate $c_1$ is merged to slice $S_1$. Gates $g_1,g_2,c_2$ are repeatedly merged to $S_2$ and gate $c_3$ is merged from $S_4$ to $S_3$. Hence every full- or half-adder logic is contained within one slice.

*Promote* In some multiplier architectures it happens the inputs of a node *g* are contained in the same slice and all three nodes are carries. In this case we decrease the number of carries by promoting *g* to the next bigger slice. More precisely we search for nodes *g* in a slice $S_{i-1}$ which have exactly one parent contained in a larger slice $S_j$ with $j \ge i-1$. If *g* would also be an input of a node in $S_{i-1}$, we cannot move *g* to slice $S_i$ without violating the topological order. The inputs of *g* also have to be contained in $S_{i-1}$ and need to have at least one parent in a bigger slice $S_j$ with $j > i-1$, i.e., they are carries. Then we promote *g* to slice $S_i$ and thus decrease the number of carries. Promoting is shown on the right side of Fig. 6 for $j=i$.

A node *g* which is merged can not be promoted back in the next round, because merging and promoting have different requirements for the input nodes of *g*. This prevents an endless alternate application of the above rules.

We can overcome the necessity of merging gates by defining slices based on the output cones of the partial products, i.e., gates which depend on a partial product. This approach works only if the partial products are generated by a simple AND-gate. If for example Booth encoding of partial products is applied we cannot identify all partial products in the AIG and thus cannot apply the approach of defining slices based on the output cones.

$$\begin{aligned} O_{i} :=\{\text{ gate } g \mid g \text{ is } \text{ in } \text{ output } \text{ cone } \text{ of } \text{ a } \text{ partial } \text{ product } a_kb_l \text{ with } k+l=i \} \end{aligned}$$

We derive slices $S_i$ as the difference of consecutive cones $O_i$:

$$\begin{aligned} S_{n-2} := O_{n-2} \qquad S_{i} := O_{i}{\setminus } \bigcup _{j=i+1}^{n-2} S_j \end{aligned}$$

The disadvantage of this approach is that the slice $S_{n-2}$ actually contains two output bits, namely $s_{n-2}$ and $s_{n-1}$. In an AIG the output bit is usually introduced by a relation of the form $s=g_k$, i.e., renaming of a gate variable $g_k$. To solve the issue we simply define a slice $S_{n-1}$ which contains exactly the relation $s_{n-1} = g_k$ for some $g_k$. This constraint is removed from $S_{n-2}$.

It can be seen in Fig. 6 that slicing based on the output cones makes the concept of merging superfluous. The node *g* in slice $S_i$ has inputs *q* and *r*, which belong to smaller slices $S_j$ and $S_k$. Hence *g* depends on the partial products of *q* and *r*. Thus *g* is in the same output cone than its children and it will be allocated to $S_l$, with $l=\text{ max }(j,k)$. So it cannot belong to a different slice.

In contrast to merging, promoting a node is still necessary, because as it can be seen in the right side of Fig. 6, nodes *g*, *q*, *r* all depend on the same partial products, hence they will all be allocated to $S_{i-1}$, which makes promoting of *g* to $S_i$ still necessary. Since promoting is necessary in both approaches and slicing based on the input cones also works for encodings, such as Booth encoding, we will stick to the slicing based on input cones.

After merging and promoting, the allocation of nodes to a slice is fixed. The slices are totally ordered starting from $S_{2n-1}$ to $S_0$. We order the nodes in a slice according to their level seen from the circuit inputs. Ordering the nodes after merging and slicing ensures that the variables are topologically sorted.

The rewriting techniques of Sect. 4 are applied in the order: Adder-Rewriting, XOR-Rewriting and Common-Rewriting. Since the structures of full- and half-adders usually do not change within a certain circuit, we do not have to compute the Gröbner basis $H_B$, cf. Sect. 5, every time we find a certain full- or half-adder structure in the corresponding AIG. The polynomials describing the adder will always have the same form. Thus it suffices that we know the structure of the polynomials in $H_B$ and simply replace the polynomials describing the adder structure by the polynomials of $H_B$ with appropriate variables. The same applies to structures describing an XOR- or XNOR-gate.

In order to simulate Common-Rewriting, we search in each slice $S_i$ for nodes which are not used in another slice and have exactly one parent. We collect them in the set $U_i$. Polynomials of nodes in $S_i$ which depend on nodes in $U_i$ are reduced first by the polynomials of nodes in $U_i$, thus eliminating the nodes of $U_i$.

After rewriting $S_i$, we search for Vanishing Constraints in the remaining nodes of $S_i$. More precisely we search for products which always evaluate to zero, e.g., *gb* in Example 1. We store these constraints in a set $V_i$ and during remainder computation we also reduce against elements of $V_i$. Since these constraints are contained in the ideal *I*(*C*), and because of Theorem 2, we can add these polynomials to the Gröbner basis without violating the Gröbner basis property.

Partial Product Elimination is handled internally. We search for all $n^2$ nodes which define a partial product in the AIG and check if they are correct. We exclude the original inputs from the AIG and treat these nodes as new inputs of the AIG. In the printing process we simply rewrite the specification in terms of these nodes.

The polynomials of each slice together with computation instructions for the incremental checking algorithm are written to a file which can be passed on to the computer algebra systems Mathematica or Singular. Whereas Singular treats the polynomials of the sliced Gröbner bases as a set which is then ordered internally according to the given variable order, it seems that Mathematica actually treats the set of polynomials as a list. Therefore it is necessary to print the polynomials in the correct order. We did not obey this fact in [28], where we actually printed the polynomials in reverse order. We started by printing the polynomials defining the partial products and ended by printing the polynomial representation of the output bit of each slice. By adjusting the printing order of the polynomials such that the leading terms of the polynomials are ordered according to the given variable order we were able to improve our computation results from [28].

## Experiments

In our work we focus on integer multipliers, as the authors of [15, 30, 31, 35], which take two *n*-bit vectors as inputs and return a bit-vector of size 2*n*. In the work of [15, 35] the authors used clean CSA multipliers, crafted from [22]. They further used multipliers generated by ABC [3] on which synthesis and technology mapping is applied. These multipliers are extremely hard to verify [15, 35].

**Fig. 7:**
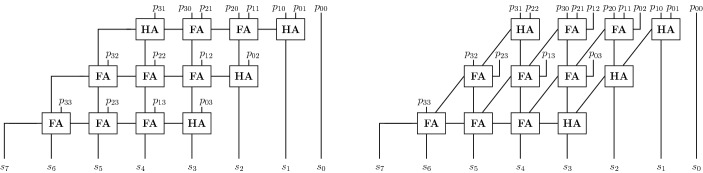
Multiplier architectures of “btor” (left) and “sp-ar-rc” (right) for input bit-width $n=4$

In our experiments we focus on two different architectures, called “btor” and “sp-ar-rc”. The “btor” benchmarks are generated from Boolector [25] and can be considered as clean multipliers. The “sp-ar-rc” multipliers are part of the bigger AOKI benchmarks [19] and can be considered as dirty multipliers. The AOKI benchmark set was used extensively in the experiments of [30, 31]. The structure of “btor” and “sp-ar-rc” multipliers is shown in Fig. 7. Both architectures can be fully decomposed into full- and half-adders, which are then accumulated. In “btor” these full- and half-adders are accumulated in a grid-like structure, whereas in “sp-ar-rc” full- and half-adders are accumulated diagonally.

In addition to “btor” and “sp-ar-rc” multipliers, we will further use more complex multiplier architectures of [15, 35] and of the AOKI benchmarks. The architectures of the different AOKI benchmarks are indicated by the names of the multipliers. The naming of the multipliers follows the following structure: “partial product generation - accumulation - last step adder”, e.g., a “sp-ar-rc” multiplier consists of simple partial product generation, which are accumulated in an array structure and the adder in the last accumulation step is a ripple-carry adder. In our experiments we will include “bp-ar-rc”, “sp-ar-cl” and “sp-wt-rc”, where *bp* defines booth encoding [26], *cl* defines a carry-lookahead adder and *wt* means accumulation by a Wallace-tree structure, where the number of partial products is reduced as soon as possible, which minimizes the overall delay of the multiplier [26].

Furthermore we use benchmarks which are synthesized and technology mapping is applied. The basis of these benchmarks is an “abc”-multiplier, which is generated with ABC [3] and has the same clean structure as the “btor” benchmarks. The different versions of synthesis and technology mapping should be the same as in [15, 35].

In all our experiments we used a standard Ubuntu 16.04 Desktop machine with Intel i7-2600 3.40 GHz CPU and 16 GB of main memory. The (wall-clock) time limit was set to 1200 s and main memory was limited to 14 GB. We measure the time from starting our tool AigMulToPoly until Mathematica resp. Singular are finished. This includes the time our tool AigMulToPoly needs to generate the files for the computer algebra system, which in the worst case is around 3 s for 128 bit multipliers. The results also include the time to launch Mathematica resp. Singular. We mark unfinished experiments by TO (reached the time limit), MO (reached the memory limit) or by an error state EE (reached the maximum number of ring variables in Singular). Singular has a limit of 32,767 on the number of ring variables and multipliers of larger bit-width easily exceed this limitation. We also mark some unfinished experiments by TO*, in this case the time limit was set to 36,000 s (10 h). Experimental data, benchmarks and source code is available at http://fmv.jku.at/fmsd18.

**Table 1:** Our column-wise incremental approach (+inc +col) versus a non-incremental approach using column-wise (−inc +col) and row-wise order (−inc +row) without Adder-Rewriting

mult	*n*	Mathematica			Singular		
		+inc	−inc+col	−inc +row	+inc	−inc+col	−inc +row
btor	16	2	3	4	1	1	2
btor	32	14	56	106	10	42	42
btor	64	131	MO	MO	MO	MO	MO
btor	128	TO	TO	TO	EE	EE	EE
sp-ar-rc	16	4	9	11	1	TO	TO
sp-ar-rc	32	30	326	425	28	TO	TO
sp-ar-rc	64	300	MO	MO	MO	MO	MO
sp-ar-rc	128	TO	TO	TO	EE	EE	EE

In Table 1 we compare our incremental column-wise verification approach of Algorithm 2 to a non-incremental verification approach, where the complete word-level specification (Definition 5) is reduced. For the non-incremental approach we use a column-wise as well as row-wise term ordering. In Table 1 all optimizations are enabled (XOR-Rewriting, Common-Rewriting, Vanishing Constraints, Merge, Promote), but Adder-Rewriting is disabled. The results show that our incremental verification approach is faster and uses less memory than the non-incremental approaches. In the experiments of [28] Mathematica needed a lot more time than Singular, but as discussed at the end of Sect. 9 we could improve the running time of Mathematica by adjusting the printing order. Hence in the experiments presented in this work the computation time of Mathematica and Singular is nearly the same. The big difference between the two computer algebra systems is that Singular needs a lot of memory, verification of 64-bit multipliers needs more than 14 GB. As expected we get an error state for the 128-bit multipliers.

**Table 2:** Effect of turning off optimizations XOR-Rewriting (−xor), Common-Rewriting (−com) and Vanishing Constraints (−vc) keeping Adder-Rewriting disabled

mult	*n*	Mathematica				Singular			
		+inc	−xor	−com	−vc	+inc	−xor	−com	−vc
btor	16	2	5	2	3	1	1	1	1
btor	32	14	31	4	15	10	28	5	12
btor	64	131	292	22	128	MO	MO	MO	MO
btor	128	TO	TO	186	TO	EE	EE	EE	EE
sp-ar-rc	16	4	17	TO	4	1	6	TO	1
sp-ar-rc	32	30	171	TO	31	28	242	TO	28
sp-ar-rc	64	300	TO	TO	303	MO	EE	MO	MO
sp-ar-rc	128	TO	TO	TO	TO	EE	EE	EE	EE

By default the adapted optimizations XOR-Rewriting, Common-Rewriting and adding Vanishing Constraints of [30] are enabled in our incremental column-wise checking algorithm. In the experiments shown in Table 2 we show the effects of turning off exactly one of these optimizations (keeping Adder-Rewriting disabled). For the “btor” multipliers turning off Common-Rewriting actually speeds up computation time. In the “btor” multipliers only a few gates with only one parent exist and applying common-rewriting by splitting remainder computation increases the run-time. In “sp-ar-rc” multipliers turning off Common-Rewriting increases computation-time drastically, because structures containing nodes with only one parent occur much more frequently. Turning off XOR-Rewriting is a downgrade for both clean and dirty multipliers. Because of the additional number of gates we already reach an error state for a 64-bit multiplier in Singular. In [28] turning off Vanishing Constraints had a very bad effect for clean multipliers in Mathematica. By printing the polynomials in a different order we could overcome this issue. Now turning off Vanishing Constraints does not influence the behavior of neither Mathematica nor Singular for clean as well as dirty multipliers. Hence the question can be asked if adding Vanishing Constraints in the current form is really necessary. Summarized it can be said that the optimizations have both positive and negative effects, depending on the structure of the multiplier.

**Table 3:** Effect turning off Merge (−merge) and Promote (−prom). Furthermore the effect of using slicing based on the output cones (+ocone)

mult	*n*	Mathematica				Singular			
		+inc	−merge	−prom	+ocone	$+$inc	−merge	−prom	+ocone
btor	16	2	3	2	3	1	1	1	1
btor	32	14	21	15	15	10	10	10	11
btor	64	131	233	133	132	MO	MO	MO	MO
btor	128	TO	TO	TO	TO	EE	EE	EE	EE
sp-ar-rc	16	4	4	TO	4	1	1	TO	1
sp-ar-rc	32	30	39	TO	31	28	29	MO	28
sp-ar-rc	64	300	430	TO	301	MO	MO	MO	MO
sp-ar-rc	128	TO	TO	TO	TO	EE	EE	EE	EE

In the experiments shown in Table 3 we investigate the effects of turning off the engineering methods Merge and Promote. The computation time of disabling Merge can considered to be the same. The difference can be seen in the size of $C_i$ in the log-files, e.g., in sp-ar-rc-8 the maximum number of monomials in any $C_i$ is 38, whereas in the approach with Merge enabled the maximum number is 8. Furthermore all $C_i$ are linear. Turning off Promote does not affect “btor”-multipliers but really slows down computation time of “sp-ar-rc” multipliers. Furthermore we compare our incremental slicing based on the input cones to the slicing method which is based on the output cones. Both slicing approaches lead to identical output files for the computer algebra systems, hence we have the same computation time in both approaches.

**Table 4:** Enabling Adder-Rewriting and partial product elimination

mult	*n*	Mathematica					Singular				
		+inc		+Adder Rew.			+inc		+Adder Rew.		
			+as		+ppe	−s		+as		+ppe	−s
btor	16	2	2	1	1	0	1	0	1	0	0
btor	32	14	15	2	2	2	10	11	1	1	1
btor	64	131	132	11	6	5	MO	MO	14	9	5
btor	128	TO	TO	101	47	40	EE	EE	EE	EE	EE
sp-ar-rc	16	4	4	1	1	1	1	1	0	0	0
sp-ar-rc	32	30	30	2	2	1	28	28	2	1	1
sp-ar-rc	64	300	295	11	6	5	MO	MO	16	10	5
sp-ar-rc	128	TO	TO	102	48	41	EE	EE	EE	EE	EE

In Table 4 we apply Adder-Rewriting on top of our incremental verification approach. In the first step we simply add the full- and half-adder specifications (+as) to the Gröbner basis, without eliminating any internal variable. Comparing the computation time, it seems that computer algebra systems cannot use this additional redundant information, similar to Vanishing Constraints in Table 2. Applying Adder-Rewriting by eliminating internal variables in the sliced Gröbner bases has a tremendous effect on the computation time. Now also 128-bit multipliers can be verified within roughly 100 s, while before verification timed out after 20 min. Additionally eliminating the partial products (+ppe) further speeds-up computation time. We assume that the considered multipliers are correct and since they can fully be decomposed into full- and half-adders, we never have to reduce by the sum output of a full- or half-adder separately. It is always reduced in parallel with the carry output. Elimination of the polynomials where the leading term is a sum-output of an adder from the Gröbner basis (−s) brings further improvements, but loses completeness.

**Table 5:** Complex multiplier architectures, including synthesis and technology mapping

mult	*n*	Mathematica	Singular
abc-resyn3-no-comp	4	1	0
abc-resyn3-no-comp	8	2	7
abc-resyn3-no-comp	16	TO	TO
abc-resyn3-comp	4	1	0
abc-resyn3-comp	8	TO	TO
bp-ar-rc	4	TO	287
bp-ar-rc	8	TO	TO
sp-ar-cl	4	1	1
sp-ar-cl	8	TO	TO
sp-wt-rc	4	1	1
sp-wt-rc	8	2	1
sp-wt-rc	16	TO	TO

In the experiments shown in Table 5 we consider the more complex multiplier architectures introduced at the beginning of this section. We apply our default incremental-checking approach without Adder-Rewriting, because usually the regular full- and half-adder structures are destroyed by synthesis and technology mappings. Synthesizing and application of complex mappings makes it very hard to verify a circuit. Even an 8-bit multiplier cannot be verified any more, neither in Mathematica nor in Singular. This confirms the results of [15, 35]. It can further be seen that more complex architectures cannot be verified with the state-of-the-art approach, which makes more sophisticated reasoning necessary.

**Table 6:** Incremental column-wise equivalence checking with and without Adder-Rewriting

mult	*n*	Lingeling [5]	ABC [3]	−Adder Rew.	+Adder Rew.
btor versus sp-ar-rc	8	14	12	2	1
btor versus sp-ar-rc	16	TO*	TO*	6	1
btor versus sp-ar-rc	32	–	–	44	3
btor versus sp-ar-rc	64	–	–	443	15
btor versus sp-ar-rc	128	–	–	TO	115

In the experiments of Table 6 we apply the column-wise equivalence checking algorithm of Sect. 8 and check the equivalence of the “btor” and “sp-ar-rc” multipliers. Despite their architectural similarities neither Lingeling [5] nor ABC [3] are able to verify their equivalence for $n=16$ within 10 h, whereas it takes only around a second using our approach based on computer algebra. In this experiment we only use Mathematica as a computer algebra system, because it supports more variables. We check the equivalence using our incremental equivalence checking algorithm with and without Adder-Rewriting. Enabling Adder-Rewriting again substantially reduces computation time. We do not use Partial Product Elimination, because in this setting we would have to manually map the AND-gates which generate the partial products of the two multipliers.

## Conclusion

This article presents in detail our incremental column-wise verification approach to formally verify integer multiplier circuits, as introduced in [8, 28, 29].

We give a precise mathematical formalization of the theory of arithmetic circuit verification using computer algebra including rigorous soundness and completeness arguments. Our incremental column-wise checking algorithm has tremendously positive effects on computation time. We discuss several optimizations which rewrite and simplify the Gröbner basis. For these optimizations we introduce the necessary theory and present a technical theorem which allows to rewrite local parts of the Gröbner basis based on [8]. Furthermore we show how our incremental verification algorithm can be extended to equivalence checking [29]. As a novel contribution we revise our engineering techniques and present a simple alternative method to define column-wise slices. We further improve computation times compared to [28] by changing the printing process of our tool.

As future work, we want to extend our methods to more complex architectures, i.e., we want to efficiently verify multiplier architectures used in Table 5. We also want to consider overflow-semantics and negative numbers. Furthermore we want to investigate floating points and other word-level operators.
